# Secreted gelsolin inhibits DNGR-1-dependent cross-presentation and cancer immunity

**DOI:** 10.1016/j.cell.2021.05.021

**Published:** 2021-07-22

**Authors:** Evangelos Giampazolias, Oliver Schulz, Kok Haw Jonathan Lim, Neil C. Rogers, Probir Chakravarty, Naren Srinivasan, Oliver Gordon, Ana Cardoso, Michael D. Buck, Enzo Z. Poirier, Johnathan Canton, Santiago Zelenay, Stefano Sammicheli, Natalia Moncaut, Sunita Varsani-Brown, Ian Rosewell, Caetano Reis e Sousa

**Affiliations:** 1 Immunobiology Laboratory, The Francis Crick Institute, 1 Midland Road, London NW1 1AT, UK; 2 Department of Immunology and Inflammation, Imperial College London, Du Cane Road, London W12 0NN, UK; 3 Bioinformatics and Biostatistics, The Francis Crick Institute, 1 Midland Road, London NW1 1AT, UK; 4 Genetic Modification Services, The Francis Crick Institute, 1 Midland Road, London NW1 1AT, UK

**Keywords:** DNGR-1, CLEC9A, dendritic cells, secreted gelsolin, cross-presentation, F-actin, cancer immunity

## Abstract

Cross-presentation of antigens from dead tumor cells by type 1 conventional dendritic cells (cDC1s) is thought to underlie priming of anti-cancer CD8^+^ T cells. cDC1 express high levels of DNGR-1 (a.k.a. CLEC9A), a receptor that binds to F-actin exposed by dead cell debris and promotes cross-presentation of associated antigens. Here, we show that secreted gelsolin (sGSN), an extracellular protein, decreases DNGR-1 binding to F-actin and cross-presentation of dead cell-associated antigens by cDC1s. Mice deficient in *sGsn* display increased DNGR-1-dependent resistance to transplantable tumors, especially ones expressing neoantigens associated with the actin cytoskeleton, and exhibit greater responsiveness to cancer immunotherapy. In human cancers, lower levels of intratumoral *sGSN* transcripts, as well as presence of mutations in proteins associated with the actin cytoskeleton, are associated with signatures of anti-cancer immunity and increased patient survival. Our results reveal a natural barrier to cross-presentation of cancer antigens that dampens anti-tumor CD8^+^ T cell responses.

## Highlights

• Secreted gelsolin (sGSN) inhibits DNGR-1 binding to F-actin
• sGSN dampens DNGR-1-dependent cross-presentation of dead cell-associated antigens
• sGSN impairs DNGR-1-dependent cDC1-mediated anti-tumor immunity
• Low sGSN expression and mutations in FABPs correlate with cancer patient survival

## Graphical abstract

**Figure:**
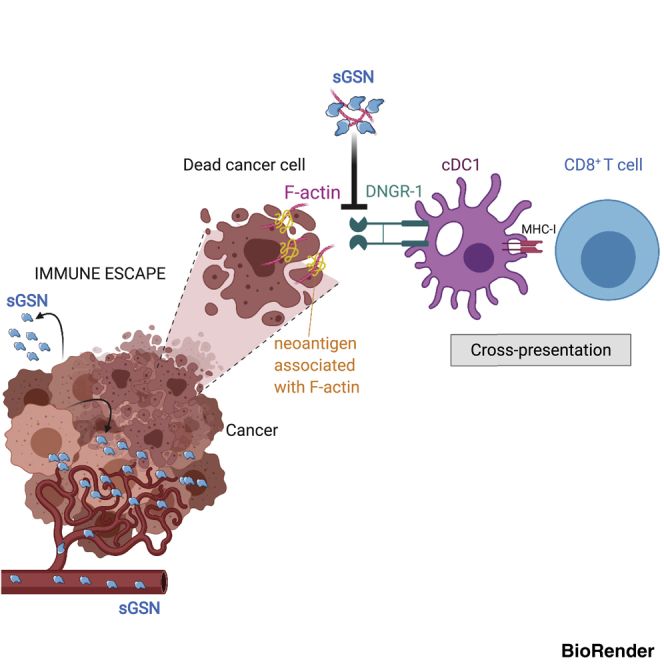

## Teaser

The secreted gelsolin component of the plasma actin-scavenging system impairs the ability of the receptor DNGR-1 to recognize dead cells and selectively dampens cross-presentation of tumor antigens by type 1 dendritic cells, acting as a barrier to anti-tumor immunity.

## Introduction

Type 1 conventional dendritic cells (cDC1s) are indispensable for effective anti-tumor immunity (Wculek et al., 2020). In mouse pre-clinical models, absence of cDC1s prevents CD8^+^ T cell-driven regression of immunogenic tumors and curtails therapeutic responses to adoptive T cell transfer or checkpoint blockade inhibition (Broz et al., 2014; Hildner et al., 2008; Salmon et al., 2016; Sánchez-Paulete et al., 2016; Spranger et al., 2017). In human cancers, cDC1 abundance correlates with CD8^+^ T cell infiltration and increased overall patient survival, as well as with clinical responses to checkpoint blockade immunotherapy (Barry et al., 2018; Böttcher et al., 2018; Michea et al., 2018). Prevention of cDC1 recruitment into the tumor microenvironment (TME) has emerged as a means of cancer immune evasion (Böttcher et al., 2018; Spranger et al., 2015; Zelenay et al., 2015). Conversely, strategies to increase the recruitment, survival, expansion, and functionality of cDC1s in the TME enhance tumor immune control and show promise as immunotherapies (Böttcher et al., 2018; Broz et al., 2014; Salmon et al., 2016; Sánchez-Paulete et al., 2018; Spranger et al., 2017; Zelenay et al., 2015). The key role of cDC1s in anti-tumor immunity is in part attributed to their ability to transport tumor antigens to draining lymph nodes and prime cancer-specific CD8^+^ T cells (Alloatti et al., 2017; Broz et al., 2014; Roberts et al., 2016; Salmon et al., 2016; Spranger et al., 2017; Theisen et al., 2018). This requires the acquisition of those antigens from tumor cells and their subsequent presentation by MHC class I molecules, a process termed cross-presentation. However, the mechanisms by which cDC1s acquire tumor antigens for cross-presentation remain unclear, and it is not known whether interference with this process can constitute a means of cancer immune evasion.

A possible source of tumor antigens for cross-presentation is necrotic cell debris (Galluzzi et al., 2017; Yatim et al., 2017), which areavidly internalized by cDC1s (Iyoda et al., 2002; Schulz and Reis e Sousa, 2002). cDC1 express high levels of the C-type lectin receptor DNGR-1 (a.k.a. CLEC9A), which binds to F-actin exposed on necrotic cell corpses (Hanč et al., 2016a) and signals post-uptake to promote cross-presentation of dead cell-associated antigens (Canton et al., 2021). Indeed, DNGR-1 signaling in ligand-containing phagosomes promotes phagosomal membrane rupture and release of antigenic material into the cytosol of cDC1s, where it can enter the endogenous MHC class I presentation pathway (Canton et al., 2021). Consistent with that finding, DNGR-1 contributes to effective CD8^+^ T cell responses to several cytopathic viruses and to allografts (Balam et al., 2020; Iborra et al., 2012, 2016; Zelenay et al., 2012). Interestingly, high *CLEC9A* expression in the TME associates with favorable prognosis in human cancer (Böttcher et al., 2018) but whether DNGR-1 plays a role in anti-tumor immunity and if it can be subverted for immune escape is not known.

Serum and plasma of all mammals contain two abundant actin-binding proteins (ABPs), secreted gelsolin (sGSN) and Gc globulin, that are thought to contribute to the removal of potentially pathological actin filaments released from or exposed by necrotic cells following tissue damage (Hartwig and Kwiatkowski, 1991; Stossel et al., 1985; Pollard and Cooper, 2003). In this so-called plasma actin-scavenging system, sGSN binds to F-actin in a Ca^2+^-dependent manner and severs the filaments for subsequent depolymerization, which is facilitated by Ca^2+^-independent sequestering of monomeric G-actin by Gc (Haddad et al., 1990; Lee and Galbraith, 1992; Lind et al., 1986; Meier et al., 2006; Vasconcellos and Lind, 1993). All cells make cytoplasmic GSN, which is an important intracellular regulator of actin filament dynamics (Kwiatkowski, 1999; Sun et al., 1999). Cells can additionally produce and secrete sGSN (Kwiatkowski et al., 1988b) by making use of an alternatively spliced exon in the *GSN* gene that encodes a signal peptide (Kwiatkowski et al., 1988a, 1986). It is reported that human cancer cells can secrete large amounts of sGSN, leading to extracellular concentrations in the TME of up to 400 μg/mL (Asare-Werehene et al., 2020; Chen et al., 2017; Tsai et al., 2012), higher than the normal circulating levels in plasma of 150–300 μg/mL (Smith et al., 1987). Cancer cell secretion of sGSN is associated with immune escape through a poorly defined mechanism (Asare-Werehene et al., 2020; Chen et al., 2017).

Here, we report that sGSN blocks DNGR-1 ligand binding and that mice selectively lacking sGSN display DNGR-1- and CD8^+^ T cell-dependent control of several transplantable tumors, especially ones expressing neoantigens associated with actin cytoskeleton. In cancer patients, lower expression of *sGSN* in the TME correlates with patient survival, especially in subcohorts of patients with increased *CLEC9A* intratumoral expression and prevalence of mutations in proteins associated with actin cytoskeleton. Collectively, our data identify sGSN as an endogenous factor that contributes to cancer immune evasion by dampening DNGR-1-dependent cross-presentation of dead cell-associated antigens by cDC1.

## Results

### sGSN inhibits DNGR-1 binding to F-actin

**Figure 1:**
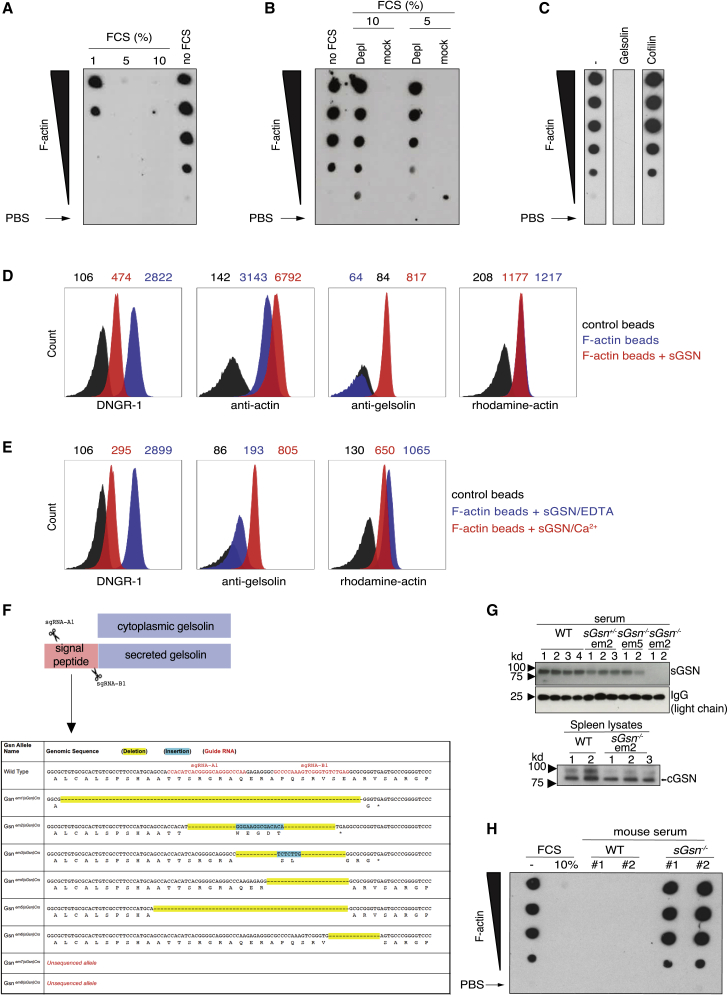
sGSN inhibits DNGR-1 binding to F-actin (A–C) Serial (2-fold) dilutions (wedge) of *in vitro* polymerized F-actin (top concentration 0.2 μM) or no F-actin (PBS; arrows) were spotted onto a membrane. DNGR-1 ECD (5 μg/mL) binding to the dots was detected following pre-treatment of the membrane with (A) the indicated doses of FCS, (B) ABP-depleted or mock-depleted FCS, and (C) sGSN or cofilin (both at 10 μg/mL). (D, and E) Flow cytometric analysis of bead-bound F-actin treated or not with (D) 10 μg/mL sGSN or (E) 10 μg/mL sGSN in the presence or absence of Ca^2+^ before staining with DNGR-1 ECD, anti-GSN, or anti-actin antibodies. Numbers above graphs represent mean fluorescence intensity for each of the three samples. (F) Generation of *sGsn*^*−/−*^ mice using CRISPR/Cas9 technology and sgRNA pairs that target the signal peptide sequence. Table shows different enzymatically modified (em1–8) mutant alleles generated and their predicted protein sequence. (G) Serum (top panel) and spleen lysates (bottom panel) from intercrossed littermate mutant mice genotyped for the indicated alleles were immunoblotted for the indicated proteins. WT indicates mice that after genotyping were deemed *sGsn*^*+/+*^. Homozygous line em2 was selected for further characterization and is henceforth referred to as *sGsn*^*−/−*^ mice. (H) Dot blot analysis of DNGR-1 ECD binding to immobilized F-actin, pre-treated or not with FCS or 10% mouse serum from WT or *sGsn*-deficient mice. #1 and #2 represent serum from individual mice. Data are representative of (E) two, (B, C, and H) three, and (A and D) six independent experiments. See also Figure S1.

DNGR-1 triggering by F-actin is potentiated by ABPs such as myosin II (Schulz et al., 2018). We wondered whether other ABPs might act instead as inhibitors of DNGR-1. We noticed that fetal calf serum (FCS), used instead of milk powder as a blocking reagent in a dot blot (Ahrens et al., 2012), inhibited binding of the extracellular domain of DNGR-1 (DNGR-1 ECD) to immobilized F-actin in a dose-dependent manner (Figure 1A). To assess if this involved actin-binding molecules present in FCS, we mixed the serum with F-actin and discarded the latter, together with any bound material, by high-speed centrifugation. FCS treated in this manner failed to inhibit DNGR-1 binding to immobilized F-actin (Figure 1B). Consistent with the serum factor in question being sGSN, treatment of membrane-immobilized F-actin with human recombinant sGSN completely abolished DNGR-1 binding, while treatment with cofilin, a cellular ABP that also destabilizes actin filaments (Carlier et al., 1999; Moon and Drubin, 1995) had no effect (Figure 1C). To more quantitatively measure gelsolin interference with DNGR-1 binding, we switched to flow cytometric analysis of bead-bound, fluorescent F-actin. Recapitulating the dot blot findings, binding of DNGR-1 ECD to F-actin beads was reduced in the presence of sGSN (Figure 1D). The total amount of fluorescent rhodamine-actin on beads was unchanged by sGSN incubation (Figure 1D), and binding of anti-actin antibody was unaffected or even slightly increased, perhaps due to increased exposure of epitopes (Figure 1D). The latter observation suggests that sGSN outcompetes DNGR-1 for binding to F-actin rather than simply causing loss of the ligand from beads through filament severing. As expected, binding of sGSN to bead-bound F-actin and its ability to subsequently block DNGR-1 was prevented by calcium chelation (Figure 1E).

### Generation of *sGsn*-deficient mice

**Figure S1:**
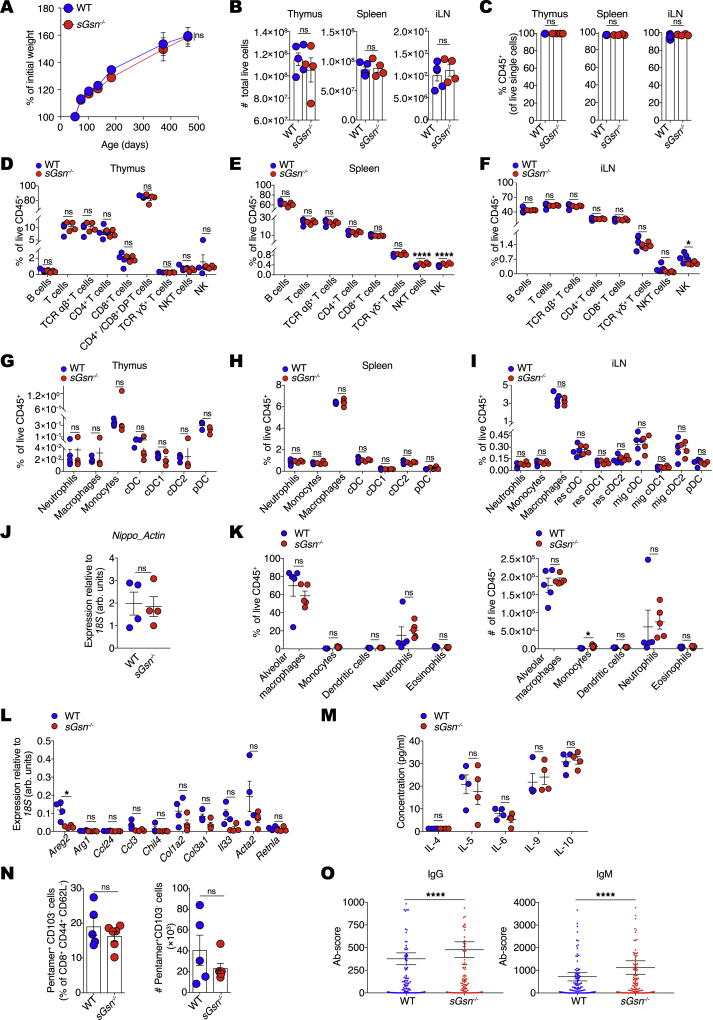
*sGsn*^*−/−*^ mice exhibit normal immune profiles, related to Figure 1 (A) Weight curves for WT (n = 5) and *sGsn*^*−/−*^ (n = 5) mice over time. (B) Cells from thymus, spleen and inguinal lymph nodes (iLN) of WT (n = 5) and *sGsn*^*−/−*^ (n = 4) mice were counted using the automated cell counter ViCell. Cell viability was measured using trypan blue exclusion. (C) The frequency of live CD45^+^ cells in thymus, spleen and iLN of WT (n = 5) and *sGsn*^*−/−*^ (n = 4) mice was measured using flow cytometry. (D–I) Flow cytometric analysis of the indicated immune cell populations in thymus, spleen and iLN of WT (n = 5) and *sGsn*^*−/−*^ (n = 4) mice. (J) WT (n = 4) or *sGsn*^−/−^ (n = 4) mice were infected subcutaneously with *N. brasiliensis*. Lungs were harvested day 3 post-infection and parasite actin mRNA levels were determined by qRT-PCR in bronchoalveolar lavage fluid (BALF) samples as a measure of infectious burden. (K) WT (n = 5) or *sGsn*^−/−^ (n = 5) mice were infected subcutaneously with *N. brasiliensis*. Flow cytometric analysis of the indicated immune cell populations in BALF samples on day 3 post-infection. Percentage of live CD45^+^ cells (left) and total numbers of indicated immune populations (right) are shown. (L and M) WT (n = 4) or *sGsn*^−/−^ (n = 4) mice were infected subcutaneously with *N. brasiliensis*. (L) Transcripts encoding of markers of type 2 immunity or (M) the indicated cytokines were measured in BALF samples. (N) Quantitation of effector memory CD8^+^ T cells in WT (n = 5) or *sGsn*^−/−^ (n = 6) mice after intranasal challenge with influenza A virus X31. Graphs show frequency (left) and numbers (right) of effector memory CD8^+^ T cells (Db-NP366-374 pentamer^+^ CD103^-^ cells) in the lungs of infected mice. (O) IgG and IgM auto-antibodies were measured in serum of aged WT (n = 5) and *sGsn*^*−/−*^ (n = 5) co-housed mice. Antibody score is shown as mean ± SEM. Data (A-O) are plotted as mean ± SEM and are representative of one experiment (A, L-O) and two experiments (B-K). Weight curves (A) were analyzed using Bonferroni-corrected two-way ANOVA. Number of cells, frequency of immune subsets, transcript expression and cytokine concetration (B-N) were compared using two-tailed unpaired t test with Welch’s correction. Auto-antibody scores (O) were compared using two-tailed Wilcoxon matched-pairs signed rank test ^∗^p ≤ 0.05, ^∗∗∗∗^p < 0.0001. ns, not significant.

By selectively targeting the alternatively spliced exon in the mouse *sGsn* locus that encodes the signal peptide, we generated C57BL/6 mice that lack secreted gelsolin (*sGsn*^−/−^) but retain cytoplasmic GSN (Figures 1F and 1G). We verified that *sGsn*^*−/−*^ mice develop and age normally (Figure S1A), as expected from the fact that total *Gsn*^*−/−*^ mice (doubly deficient in cytoplasmic GSN and sGSN) display only a mild phenotype in the C57BL/6 genetic background (Cantù et al., 2012; Witke et al., 1995). Immune profiling of *sGsn*^*−/−*^ mice revealed overall normal myeloid and lymphoid cell composition in primary and secondary lymphoid organs (Figures S1B–S1I). Consistent with a normal immunological profile, *sGsn*^*−/−*^ mice displayed no impairment in their ability to resist and respond to very distinct infectious challenges, namely parasite (*Nippostrongylus brasiliensis*) or viral (influenza A virus) infection (Figures S1J–S1N). They also showed no signs of autoimmunity, although they displayed marginally elevated levels of IgG and IgM auto-antibodies upon aging (>1 year; Figure S1O).

### sGSN reduces DNGR-1 triggering and cross-presentation of cell-associated antigen by cDC1s

**Figure 2:**
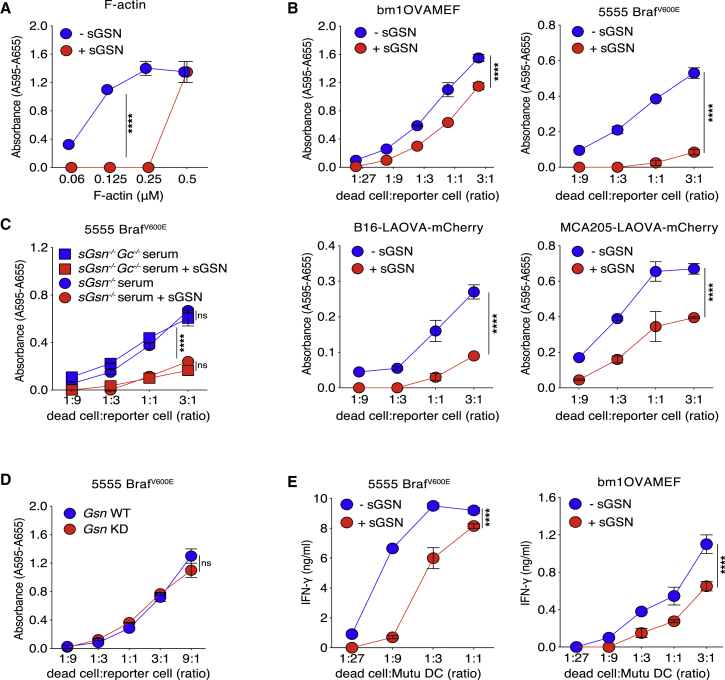
sGSN reduces DNGR-1 triggering and cross-presentation of cell-associated antigen by cDC1s (A–D) (A) Titration of F-actin or (B–D) dead cells on BWZ-mDNGR-1 reporter cells. Graphs show reporter activity measured by absorbance after addition of β-galactosidase substrate to lysed cells. Plotted data represent mean ± SD of duplicate wells. (A) F-actin in the absence or presence of added sGSN. (B) UV-treated bm1OVAMEF and tumor cell lines (5555 Braf^V600E^, B16-LAOVA-mCherry, MCA205-LAOVA-mCherry) in the absence or presence of sGSN. (C) 5555 Braf^V600E^-induced BWZ stimulation using serum from mice deficient in sGSN or doubly deficient in sGSN and Gc globulin in the absence or presence of added sGSN. (D) Comparison of UV-treated parental (expressing GSN; blue circles) and GSN knockdown 5555 Braf^V600E^ cells (lacking GSN; red circles). (E) UV-treated 5555 Braf^V600E^ (left panel) cells pulsed with OVA or bm1OVAMEF (right panel) cells were added at various doses to Mutu DC in the absence or presence of sGSN and co-cultured with pre-activated OT-I cells. Graphs show concentration of IFN-γ in the supernatant after overnight culture. Plotted data represent mean ± SD of duplicate wells. Data are representative of two (C and D) and three (A, B, and E) independent experiments. All data were analyzed using Bonferroni-corrected two-way ANOVA. ^∗∗∗∗^p < 0.0001; ns, not significant. See also Figure S2.

**Figure S2:**
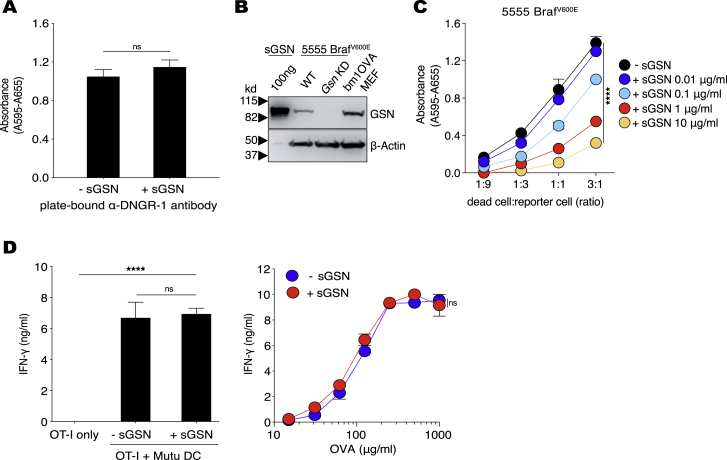
sGSN specifically inhibits DNGR-1-mediated responses to cell-associated F-actin ligand, related to Figure 2 (A and C) Stimulation of BWZ-mDNGR-1 reporter cells by plate-bound anti-DNGR-1 antibody in the absence or presence of sGSN (A) and titration of UV-treated 5555 Braf^V600E^ on BWZ-mDNGR-1 reporter cells in the presence of the indicated sGSN concentrations (C). Graphs show absorbance after addition of β-galactosidase substrate to lysed cells. Plotted data represent mean absorbance ± SD of duplicate wells. (B) Recombinant gelsolin (sGSN), *Gsn* WT (expressing PLKO.1 empty vector) or *Gsn* KD (expressing PLKO.1-*Gsn*^shRNA^) 5555 Braf^V600E^ and bm1OVAMEF cells were immunoblotted for gelsolin and β–actin. (D) Presentation of low dose (10 pM) SIINFEKL peptide (left panel) or the indicated concentrations of soluble OVA (right panel) in the absence or presence of sGSN in Mutu DC/OT-I T co-cultures. Graphs show concentration of IFN-γ in the supernatant of overnight cultures as mean ± SD of duplicate wells. Data (A-D) are representative of at least two independent experiments. Data in (A) were analyzed using two-tailed unpaired t test with Welch’s correction. Data in (D, left panel) were analyzed using Bonferroni-corrected one-way ANOVA. Data in (C and D, right panel) were analyzed using Bonferroni-corrected two-way ANOVA. ^∗∗∗∗^ (p < 0.0001), ns (not significant).

Like FCS, serum from mice also inhibits DNGR-1 binding to immobilized F-actin (Figure 1H). Notably, inhibition was lost when we used serum from *sGsn*^*−/−*^ mice (Figure 1H), indicating that circulating sGSN fully accounts for the inhibitory effect of serum on DNGR-1 binding to F-actin. To assess the impact on DNGR-1 function, we first used a reporter assay of DNGR-1 triggering (Sancho et al., 2009) and tested the effect of adding serum from sGSN-deficient mice supplemented or not with a defined amount (10 μg/mL) of recombinant sGSN (a dose at least 10-fold lower than physiological levels of plasma sGSN). In the presence of sGSN, stimulation of the reporter cells with F-actin alone did not generate a signal up until a concentration of ligand (0.5 μM) that exceeded the amount of added sGSN (0.1 μM) by 5-fold (Figure 2A), suggesting that sGSN blocks DNGR-1 binding sites on F-actin in a stoichiometric manner. To assess the impact of sGSN on DNGR-1 triggering by dead cells, we used UV-irradiated mouse embryonic fibroblasts or tumor cells (see below) as stimuli. Again, we found inhibition of DNGR-1 triggering by dead cell corpses in the presence of sGSN (Figure 2B). In contrast, the absence or presence of sGSN did not impact stimulation of reporter cells with plate-bound anti-DNGR-1 antibody (Figure S2A), excluding non-specific effects.

Unlike sGSN, the other component of the actin-scavenging system, Gc globulin, cannot bind to F-actin (Cooke and Haddad, 1989; Haddad et al., 1992; Lees et al., 1984) and is therefore unlikely to directly interfere with DNGR-1 triggering by ligand. Consistent with that notion, inhibition of dead cell-induced stimulation of the reporter cells was similar whether the assay was carried out with serum from sGSN-deficient mice or serum from mice doubly deficient in sGSN and Gc (Figure 2C). Cytoplasmic gelsolin potentially released from dead cells was also not sufficient to interfere with DNGR-binding as the reporter cells were stimulated equally by killed cells from the parental (gelsolin-sufficient) 5555 Braf^V600E^ tumor cell line and from a stable 5555 Braf^V600E^ gelsolin knockdown (KD) line (Figures 2D and S2B). This is likely a quantitative issue as cytoplasmic gelsolin released from dead cells is rapidly diluted to below 1 μg/mL, the concentration required to inhibit DNGR-1 triggering (Figure S2C). Finally, we examined the effect of sGSN on cross-presentation of dead cell-associated ovalbumin (OVA) antigen to CD8^+^ OT-I T cells by the Mutu cDC1 cell line (Fuertes Marraco et al., 2012), which expresses DNGR-1 (Hanč et al., 2016b). The OT-I response in cultures containing sGSN was significantly lower than that in sGSN-free mouse serum (Figure 2E). As controls, presentation of OVA (SIINFEKL) peptide or cross-presentation of soluble OVA protein was not affected by sGSN (Figure S2D), emphasizing the specificity of the inhibitory effect for cross-presentation of antigen derived from dead cells. We conclude that sGSN is necessary and sufficient for inhibition of dead cell recognition by DNGR-1 and for decreasing cross-presentation of dead cell-associated antigens.

### Loss of *sGsn* in mice promotes tumor resistance

**Figure S3:**
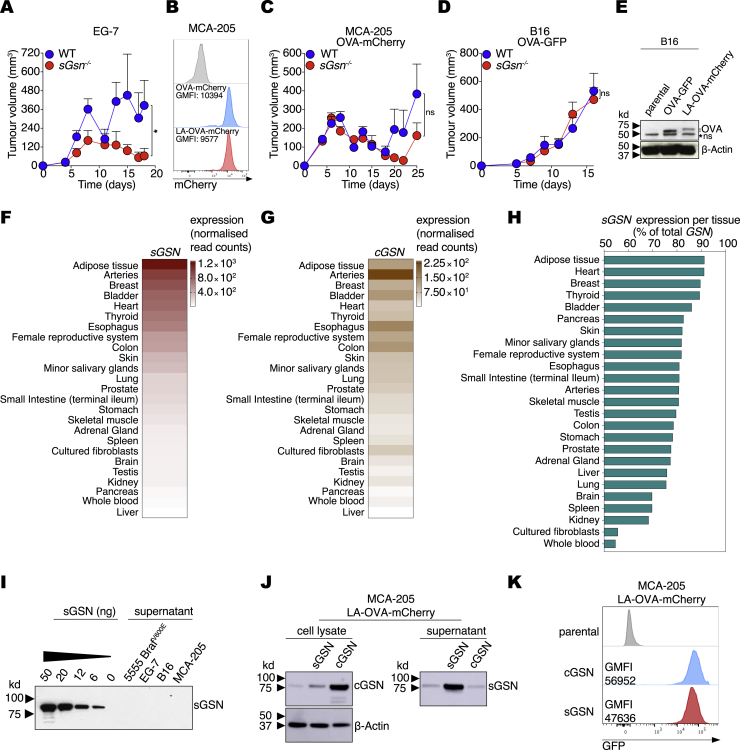
sGSN expression and impact on tumor growth, related to Figure 3 (A) Growth profile of tumors following subcutaneous inoculation of 1 × 10^6^ EG-7 cancer cells in WT (n = 8) or *sGsn*^*−/−*^ (n = 8) co-housed mice. (B) Intensity of mCherry fluorescence (geometric mean; GMFI) in MCA-205 parental cells or cells expressing either OVA-mCherry or LA-OVA-mCherry. (C and D) Growth profile of tumors following subcutaneous inoculation of (C) 0.5 × 10^6^ MCA-205 cancer cells expressing OVA-mCherry into WT (n = 9) or *sGsn*^*−/−*^ (n = 9) mice or (D) 0.3 × 10^6^ B16.F10 cancer cells expressing OVA-GFP into WT (n = 9) or *sGsn*^*−/−*^ (n = 6) mice. (E) Lysates from B16F10 parental cells or cells expressing either OVA-GFP or LA-OVA-mCherry were separated by SDS-PAGE and immunoblotted for OVA and β–Actin. ns, non-specific band. (F and G) Human tissue expression of (F) *sGSN* and (G) *cGSN* from the Genotype-Tissue Expression (GTEx) database. (H) *sGSN* isoform as a percentage of total gelsolin transcript expression in human tissues. (I) Recombinant gelsolin (sGSN) or supernatants from cultures of the indicated tumor cell lines were separated by SDS-PAGE and immunoblotted for gelsolin. (J) Cell lysates and supernatant of MCA-205 LA-OVA-mCherry tumors expressing cGSN or sGSN were separated by SDS-PAGE and immunoblotted for gelsolin and β-Actin. (K) GFP fluorescence of MCA-205 LA-OVA-mCherry tumors as surrogate for cGSN and sGSN expression. Data in (A, C, D) are mean tumor volume ± SEM and are representative of two independent experiments for A, D and one experiment for C. Tumor growth profiles (A, C, D) were compared using Bonferroni-corrected two-way ANOVA. ^∗^p ≤ 0.05, ns, not significant.

**Figure 3:**
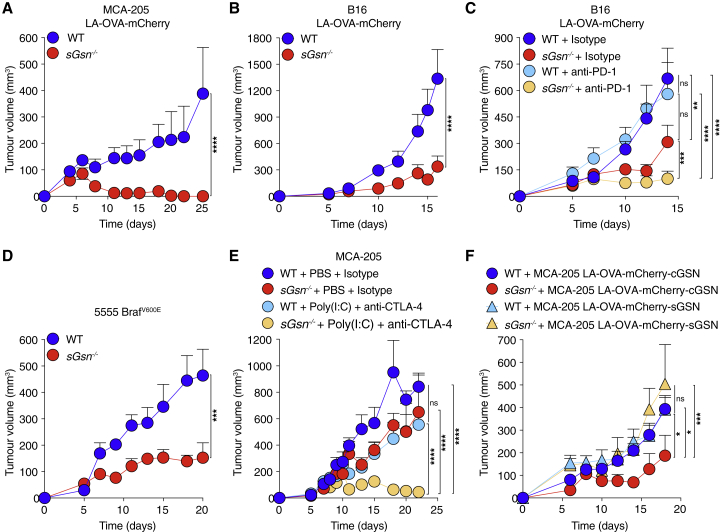
Loss of sGSN impairs tumor growth and augments response to immune checkpoint blockade (A–C) Growth profile following subcutaneous inoculation of cancer cell lines expressing LA-OVA-mCherry into WT (C57BL/6J) or *sGsn*^*−/−*^ mice. (A) 0.5 × 10^6^ MCA-205 LA-OVA-mCherry cancer cells implanted in WT (n = 10) or *sGsn*^*−/−*^ (n = 10) mice. (B) 0.3 × 10^6^ B16F10 LA-OVA-mCherry cancer cells implanted in WT (n = 10) or *sGsn*^*−/−*^ (n = 10) mice. (C) 0.3 × 10^6^ B16F10 LA-OVA-mCherry cancer cells implanted in WT or *sGsn*^*−/−*^ mice that received 200 μg of isotype control or anti-PD-1 monoclonal antibody intraperitoneally (i.p.) every 3 days from day 3 to day 14. WT + isotype (n = 10), *sGsn*^*−/−*^ + isotype (n = 9), WT + anti-PD-1 (n = 10), *sGsn*^*−/−*^ + anti-PD-1 (n = 10). (D) Growth profile of 0.2 × 10^6^ 5555 Braf^V600E^ cancer cells implanted in WT littermate control (*sGsn*^*+/+*^) mice (n = 5) and *sGsn*^*−/−*^ mice (n = 5). (E) Growth profile of 0.5 × 10^6^ MCA-205 cancer cells implanted in WT or *sGsn*^*−/−*^ mice. Mice received 50 μg of Poly(I:C) or PBS (days 7 and 11) injected intratumorally in the presence of 50 μg of isotype control or anti-CTLA-4 (days 6 and 12) injected i.p. WT + PBS + isotype (n = 6 mice), *sGsn*^*−/−*^ + PBS + isotype (n = 5 mice), WT + Poly(I:C) + anti-CTLA-4 (n = 8 mice), *sGsn*^*−/−*^ + Poly(I:C) + anti-CTLA-4 (n = 8 mice). (F) Growth profile of 0.5 × 10^6^ MCA-205 LA-OVA-mCherry cancer cells expressing either cGSN or sGSN, implanted in WT (n = 9, cGSN, n = 9, sGSN) or *sGsn*^*−/−*^ mice (n = 7, cGSN, n = 8, sGSN). Data in (A–F) are presented as tumor volume (mm^3^) ± SEM and are representative of at least two independent experiments. Tumor growth profiles (A–F) were compared using Bonferroni-corrected two-way ANOVA. ^∗^p ≤ 0.05, ^∗∗^p < 0.01, ^∗∗∗^p < 0.001, ^∗∗∗∗^p < 0.0001; ns, not significant. See also Figure S3.

As cross-presentation is a limiting factor in anti-tumor immunity (Kozik et al., 2020), we hypothesized that *sGsn*^*−/−*^ mice might display increased anti-tumor CD8^+^ T cell responses. Consistent with this possibility, highly immunogenic tumors derived from an OVA-expressing thymoma cell line (EG7) exhibited faster and increased regression in sGSN-deficient mice compared to C57BL/6 wild-type (WT) mice (Figure S3A). However, this was not universally seen with OVA-expressing tumors: a weakly immunogenic fibrosarcoma line (MCA-205) expressing OVA (lacking the signal sequence and fused to mCherry; Figure S3B) was controlled similarly in sGSN-deficient and WT mice (Figure S3C). CD8^+^ T cell responses against neoantigens associated with the actin cytoskeleton can lead to partial or complete tumor regression in both mice and humans (Matsushita et al., 2012; Zorn and Hercend, 1999). To test whether the relative tumor resistance of sGSN-deficient mice is more marked in settings in which the relevant tumor antigens are associated with the actin cytoskeleton, we fused the OVA-mCherry construct to the 17 amino acid sequence of the LifeAct F-actin binding peptide (Riedl et al., 2008) and expressed the new construct (LA-OVA-mCherry) in the same weakly immunogenic cancer cell line MCA-205 (Figure S3B). We found that *sGsn*^−/−^ mice controlled LA-OVA-mCherry MCA-205 tumors much better than WT controls (Figure 3A). Indeed, complete rejection of these tumors accompanied by remission was only seen in *sGsn*-deficient hosts (Figure 3A). Similarly, expression of LA-OVA-mCherry in the poorly immunogenic B16F10 melanoma cell line permitted tumor control preferentially in the sGSN-deficient mouse strain (Figure 3B), which, as for MCA205, was not the case with B16F10 expressing OVA not fused to the LA peptide (Figure S3D). Further analysis clearly indicated that tumor control in *sGsn*^−/−^ mice correlates with cytoskeletal association of antigen rather than antigen levels (Figures S3B and S3E). Control of LA-OVA-mCherry B16F10 tumors in *sGsn*^−/−^ mice was further enhanced by anti-PD-1 immune checkpoint blockade, which, by itself, had no effect in WT mice (Figure 3C). The tumor resistance phenotype of sGSN-deficient mice was also apparent with some unengineered tumor cell lines. This was the case with the 5555 Braf^V600E^ melanoma cell line (Dhomen et al., 2009) (Figure 3D) or even the parental MCA-205 line not expressing OVA when its immunogenicity was boosted by treating with the immune checkpoint inhibitor anti-CTLA-4 together with the immune stimulator poly(I:C) (Figure 3E). Thus, *sGsn*^−/−^ mice exhibit greater resistance to a variety of immunogenic transplantable tumors, which is especially marked for those that bear tumor neoantigens that associate with the actin cytoskeleton.

Many cells can synthesize sGSN in addition to cytoplasmic gelsolin (cGSN), and *sGSN* can account for more than half of total gelsolin transcript expression in some tissues (Figures S3F–S3H). In line with this, several human cancers have been reported to secrete sGSN (Asare-Werehene et al., 2020; Chen et al., 2017; Tsai et al., 2012), unlike the murine cancer cell lines used in this study (Figure S3I). We therefore overexpressed *sGSN* in MCA-205 LA-OVA-mCherry cells (Figure S3J) and challenged WT and *sGsn*^−/−^ mice. As a control, we overexpressed *cGSN*, ensuring equal levels of expression by means of a surrogate GFP marker (Figure S3K). Notably, forced sGSN but not cGSN expression abrogated the relative resistance of *sGsn*^−/−^ mice to LA-OVA-mCherry tumors (Figure 3F), indicating that sGSN secretion by cancer cells can function as an escape strategy.

### Increased tumor resistance of sGSN-deficient mice is due to increased DNGR-1-mediated cross-priming of antigen-specific CD8^+^ T cells

**Figure 4:**
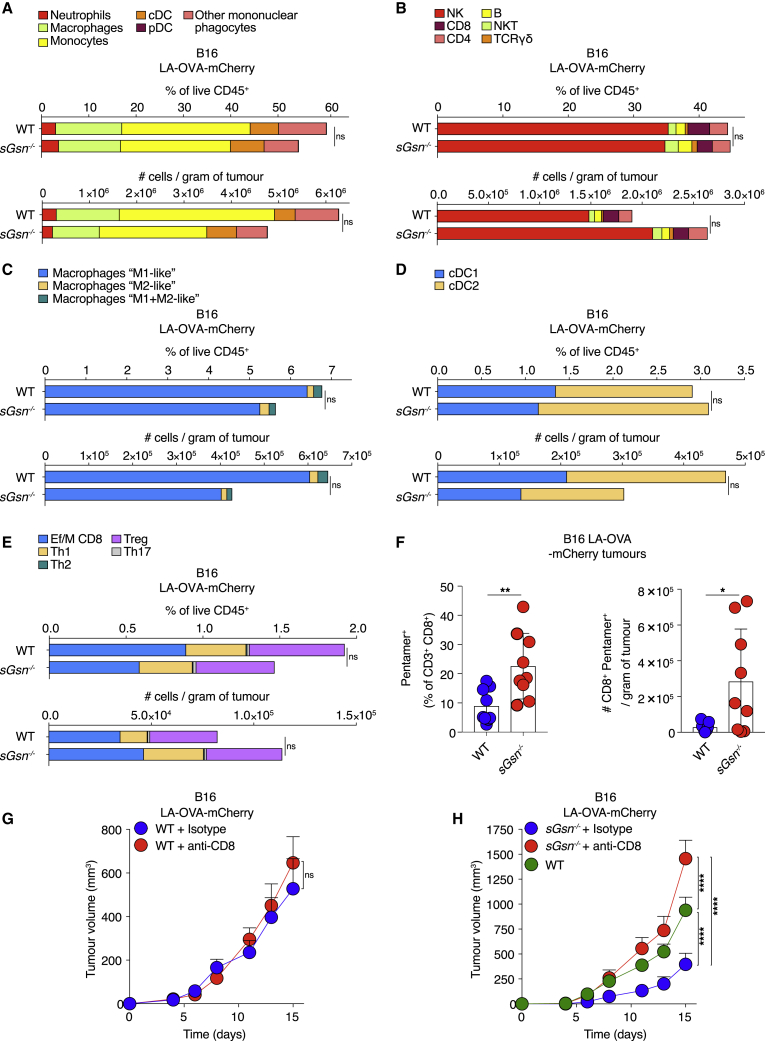
Loss of sGSN permits tumor control dependent on CD8^+^ T cells (A–E) Quantification of the indicated immune cell populations in the TME of B16 LA-OVA tumors growing in WT (n = 9) or *sGsn*^*−/−*^ (n = 10) mice at day 14 post-inoculation. Data are mean of frequency (%) of CD45^+^ cells (top) or the numbers of cells per gram of tumor (bottom) and are representative of two independent experiments. (F) Quantification of intra-tumoral CD8^+^ OVA-specific pentamer^+^ cells at day 16 following subcutaneous inoculation of 0.3 × 10^6^ B16F10 cancer cells expressing LA-OVA-mCherry into WT (n = 9) or *sGsn*^*−/−*^ (n = 9) co-housed mice. Data are mean ± SEM of frequency of OVA-specific pentamer^+^ (% of CD3^+^ CD8^+^) cells (left) or the number of CD8^+^ OVA-pentamer^+^ cells per gram of tumor (right) and are representative of two experiments. (G) Growth profile of 0.3 × 10^6^ B16F10 cancer cells expressing LA-OVA-mCherry implanted in WT mice. Mice received 300 μg of isotype control or anti-CD8 i.p. (days −3, 1, 4, 7, 10, 13). WT + isotype (n = 10) and WT + anti-CD8 (n = 10). (H) As in (G) but using *sGsn*^*−/−*^ mice and comparing to an untreated WT group. WT (n = 21), *sGsn*^*−/−*^ + isotype (n = 10) and *sGsn*^*−/−*^ + anti-CD8 (n = 10). Groups in (A–F) were compared using two-tailed unpaired t test with Welch’s correction. Tumor growth profiles (G and H) were compared using Bonferroni-corrected two-way ANOVA. ^∗^p ≤ 0.05, ^∗∗^p < 0.01, ^∗∗∗∗^p < 0.0001; ns, not significant. See also Figure S4.

**Figure S4:**
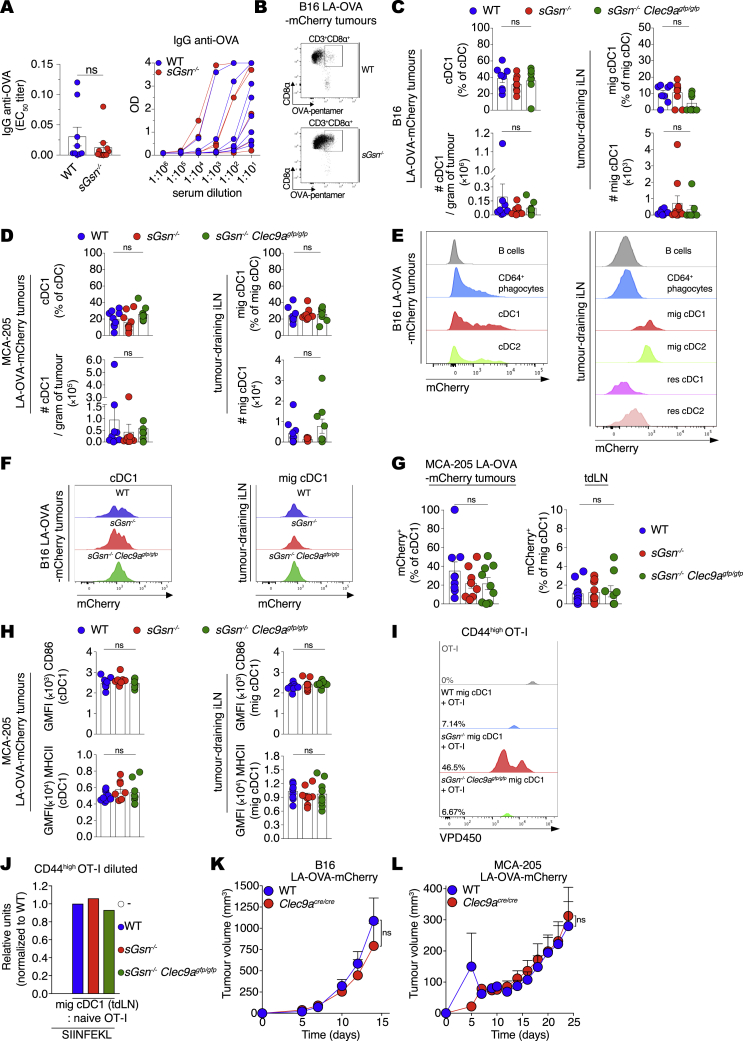
Loss of sGSN does not impact tumor antigen uptake and activation status of cDC1s, related to Figures 4 and 5 (A) OVA-specific IgG antibody response in WT and *sGsn*^*−/−*^mice injected with MCA-205 LA-OVA-mCherry cells expressing cGSN as in (Figure 3F) on day 30 post-tumor inoculation. EC_50_ titer (left) is shown as mean ± SEM from two experiments. Representative serum titrations from one experiment are shown on the right. (B) Representative dot plot and gating strategy for CD8^+^ OVA-specific pentamer^+^ cells in tumor samples at day 16 post-tumor inoculation as in Figure 4F. (C and D) Quantification of cDC1 in tumors (left) and migratory cDC1 in tdLNs (right) of WT (n = 8 or 10), *sGsn*^*−/−*^ (n = 9 or 9) or *sGsn*^*−/−*^*Clec9a*^*gfp/gfp*^ (n = 7 or 9) mice injected with (C) B16F10 LA-OVA-mCherry at day 15 or (D) MCA-205 LA-OVA-mCherry tumor cells analyzed at day 26 post-inoculation. Data (C, D) are presented as mean frequency (top) or number of cDC1 cells per gram of tumor (bottom) ± SEM and are representative of two experiments (C) and one experiment (D). (E) Representative histogram of tumor-derived mCherry across the indicated immune populations in B16F10 LA-OVA-mCherry tumors (left) and tdLNs (right) at day 15 post-inoculation. (F) Representative histograms of mCherry fluorescence in WT, *sGsn*^*−/−*^ or *sGsn*^*−/−*^*Clec9a*^*gfp/gfp*^ cDC1 or mig cDC1 intratumorally (left) and in the tdLN (right) of B16F10 LA-OVA-mCherry tumors at day 15 post-inoculation as in Figure 5A. (G) Quantification of mCherry^+^ cDC1 or mig cDC1 in WT (n = 10), *sGsn*^*−/−*^ (n = 9) or *sGsn*^*−/−*^*Clec9a*^*gfp/gfp*^ (n = 9) intratumorally (left) and in the tdLN (right) of MCA-205 LA-OVA-mCherry tumors at day 26 post-inoculation. Data are mean ± SEM and are representative of one experiment.. (H) Quantification of geometric mean fluorescent intensity of CD86 and MHC class II staining of cDC1 or mig cDC1 intratumorally (left) and in the tdLN (right) at day 26 post-tumor inoculation with MCA-205 LA-OVA-mCherry into WT (n = 9 or n = 9), *sGsn*^*−/−*^ (n = 7 or n = 9) or *sGsn*^*−/−*^*Clec9a*^*gfp/gfp*^ (n = 8 or n = 9). Data are mean ± SEM and are representative of one experiment. (I) Representative flow cytometric plot of naive OT-I proliferation as measured by dilution of VPD450 dye at 72 h following co-culture with the tdLN mig cDC1 derived from WT, *sGsn*^*−/−*^ or *sGsn*^*−/−*^*Clec9a*^*gfp/gfp*^ at day 14 post-inoculation (B16F10 LA-OVA-mCherry) as in Figure 5C. (J) Quantification of naive OT-I proliferation following *ex vivo* co-culture with sorted mig cDC1 as in Figure 5C from WT (n = 13), *sGsn*^*−/−*^ (n = 12) or *sGsn*^*−/−*^*Clec9a*^*gfp/gfp*^ (n = 10) in the presence of 10 pM SIINFEKL peptide. Data are mean of relative units (% OT-I proliferated cells normalized to WT) and are representative of one experiment. (K and L) Growth profile of tumors formed following subcutaneous inoculation of (K) 0.3 × 10^6^ B16F10 cancer cells expressing LA-OVA-mCherry implanted in WT (n = 7) or *Clec9a*^*cre/cre*^ (n = 6) co-housed mice or (L) 0.5 × 10^6^ MCA-205 cancer cells expressing LA-OVA-mCherry into WT (n = 10) or *Clec9a*^*cre/cre*^ (n = 10) co-housed mice. Groups in (C, D, G, H) were compared using Bonferroni-corrected one-way ANOVA. Tumor growth profiles (K, L) are presented as tumor volume (mm^3^) ± SEM, are representative of one experiment. and were compared using Bonferroni-corrected two-way ANOVA. ns, not significant.

The fact that *sGsn*^−/−^ mice were more responsive to cancer immunotherapy suggested an immune-dependent mechanism of resistance. Analysis of the B16F10 LA-OVA-mCherry TME did not reveal any significant differences in composition between *sGsn*^*−/−*^ and WT mice (Figures 4A–4E). There were also no differences in OVA-specific antibodies between tumor-bearing *sGsn*^*−/−*^ and WT mice (Figure S4A). However, we found a higher number and frequency of intratumoral OVA-specific (pentamer^+^) CD8^+^ T cells in *sGsn*^*−/−*^ mice (Figures 4F and S4B), indicating an enhanced antigen-specific response. Further, the observed relative tumor resistance of *sGsn*^*−/−*^ mice was abrogated by antibody-mediated CD8^+^ T cell depletion (Figures 4G and 4H). Therefore, the relative tumor resistance of *sGsn*^*−/−*^ mice appears due to an antigen-specific CD8^+^ T cell response.

**Figure 5:**
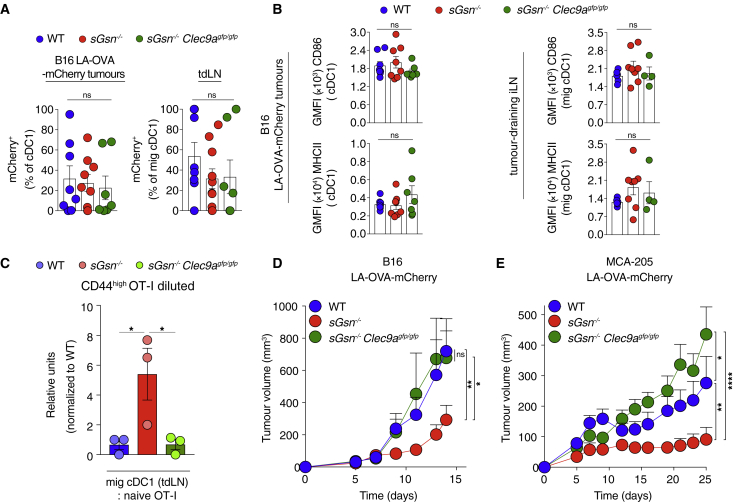
Loss of sGSN increases DNGR-1-dependent CD8^+^ T cell cross-priming by migratory cDC1s (A and B) (A) mCherry^+^ cDC1 or (B) CD86 and MHCII expression by cDC1s (geometric mean fluorescent intensity (GMFI) from tumors (left) or tdLNs (right) at day 15 post inoculation with B16F10 LA-OVA-mCherry cancer cells into (A) WT (n = 8), *sGsn*^*−/−*^ (n = 9) or *sGsn*^*−/−*^*Clec9a*^*gfp/gfp*^ (n = 7) or (B) WT (n = 8 or n = 6), *sGsn*^*−/−*^ (n = 8 or n = 8), or *sGsn*^*−/−*^*Clec9a*^*gfp/gfp*^ (n = 7 or n = 4) mice. Data are mean ± SEM of frequency of (A) mCherry^+^ (% of migratory cDC1) cells or (B) GMFI and are representative of two experiments. (C) Quantification of naive OT-I proliferation following *ex vivo* co-culture with sorted migratory cDC1s from tdLN (inguinal and axillary) of WT (n = 44), *sGsn*^*−/−*^ (n = 41), or *sGsn*^*−/−*^*Clec9a*^*gfp/gfp*^ (n = 29) mice at day 14 post-tumor (B16F10 LA-OVA-mCherry) inoculation. Data are mean of relative OT-I proliferation (normalized to proliferation with cDC1s from WT group) ± SEM and are pooled from three independent experiments. (D and E) Growth profile of (D) 0.3 × 10^6^ B16F10 or (E) 0.5 × 10^6^ MCA-205 cancer cells expressing LA-OVA-mCherry implanted in (D) WT (n = 9), *sGsn*^*−/−*^ (n = 10), or *sGsn*^*−/−*^*;Clec9a*^*gfp/gfp*^ (n = 8) or (E) WT (n = 9), *sGsn*^*−/−*^ (n = 10), or *sGsn*^*−/−*^*; Clec9a*^*gfp/gfp*^ (n = 8) mice. Data in (D and E) are presented as tumor volume (mm^3^) ± SEM and are representative of two independent experiments. Groups in (A–C) were compared using Bonferroni-corrected one-way ANOVA. Tumor growth profiles (D and E) were compared using Bonferroni-corrected two-way ANOVA. ^∗^p ≤ 0.05, ^∗∗^p < 0.01, ^∗∗∗∗^p < 0.0001; ns, not significant. See also Figure S4.

We tested the possibility that this reflected stronger DNGR-1 activity and generated additional control mice lacking both DNGR-1 and sGSN (*sGsn*^*−/−*^*; Clec9a*^*gfp/gfp*^). Previous work shows that DNGR-1 does not impact dead cell uptake by cDC1s or cDC1 differentiation, migration, or activation (Canton et al., 2021; Sancho et al., 2009; Zelenay et al., 2012). Consistent with this notion, sGSN single or sGSN-DNGR-1 double deficiency did not impact the frequency or number of cDC1 within MCA205 or B16F10 LA-OVA-mCherry tumors or in tumor-draining lymph nodes (tdLNs; Figures S4C and S4D). Using the mCherry signal as a surrogate for uptake and retention of tumor cell material, we found, as expected (Roberts et al., 2016), that it was sampled in the TME and transported to tdLNs by migratory cDC1s and cDC2s (Figure S4E). However, DNGR-1 and/or sGSN deficiency did not alter the frequency of mCherry^+^ cDC1s in tumors or of mCherry^+^ migratory cDC1s in tdLNs (Figures 5A, S4F, and S4G). Finally, we confirmed that deficiency in sGSN, irrespective of presence or absence of DNGR-1, did not affect cDC1 activation as measured by levels of CD86 and MHC class II in either TME or tdLNs (Figures 5B and S4H).

The above data are consistent with the notion that DNGR-1 is a dedicated receptor for cross-presentation of dead-cell-associated antigens, acting post-uptake to promote rupture of phagosomes and access of internalized tumor debris to the cytosolic MHC class I presentation pathway of cDC1s (Canton et al., 2021). To assess this directly, we sorted migratory cDC1s from the tdLNs of mice bearing B16F10 LA-OVA-mCherry tumors and co-cultured them with naive OVA-specific OT-I CD8^+^ T cells. Enhanced proliferation and activation of OT-I T cells was seen with migratory cDC1s from tumor-bearing *sGsn*^*−/−*^ mice but not with those from *sGsn*^*−/−*^ *Clec9a*^*gfp/gfp*^ mice (Figures 5C and S4I). As a control, migratory cDC1s from all mice stimulated OT-I equally when pulsed with OVA peptide *ex vivo* (Figure S4J). Notably, loss of *Clec9a* completely reversed the relative resistance of sGSN-deficient mice to MCA-205 and B16F10 LA-OVA-mCherry tumors (Figures 5D and 5E) but did not impact growth of the same cancers in sGSN-sufficient hosts (Figures S4K and S4L). Collectively, our data indicate a role for DNGR-1 in promoting cross-presentation of tumor antigens in the *sGsn*-deficient background, which leads to priming of anti-tumor CD8^+^ T cells that mediate cancer rejection.

### *sGSN* expression in human cancers inversely correlates with patient survival

**Figure 6:**
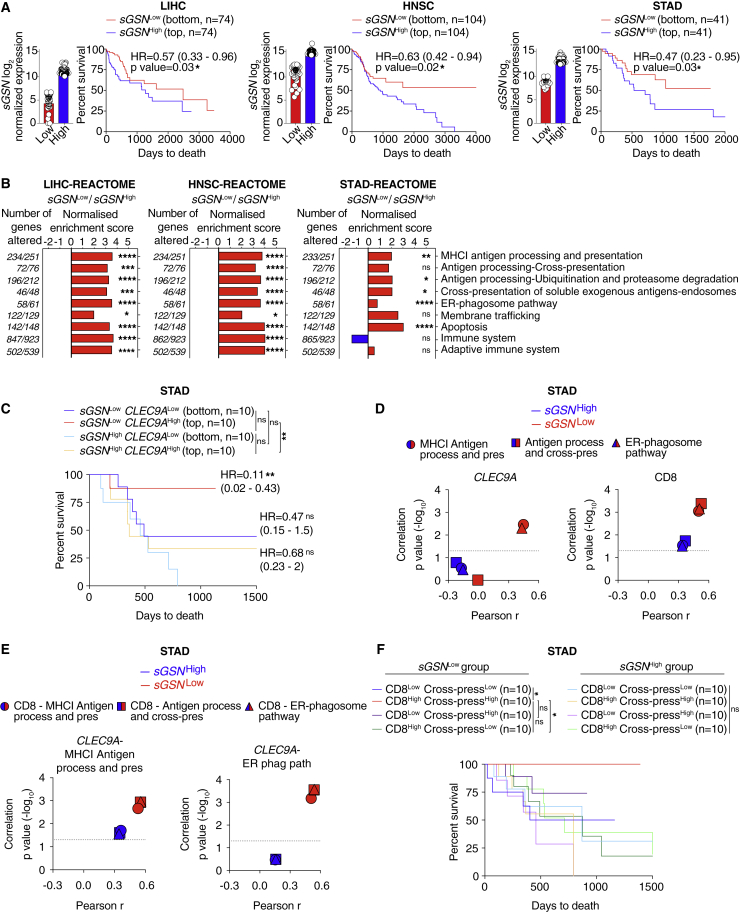
Low *sGSN* levels in human cancer biopsies correlate with survival of patients with high *CLEC9A* expression (A) Prognostic value of *sGSN* transcript levels for overall survival comparing samples with lowest (*sGSN*^Low^) and highest (*sGSN*^High^) expression in the indicated TCGA datasets. Liver hepatocellular carcinoma (LIH), bottom (n = 74) and top (n = 74) 20% of patient cohort. Head and neck squamous cell carcinoma (HNSC), bottom (n = 104) and top (n = 104) 20% of patient cohort. Stomach adenocarcinoma (STAD), bottom (n = 41) and top (n = 41) 10% of patient cohort. (B) Gene set enrichment analysis in the lowest (*sGSN*^Low^) group compared to the highest (*sGSN*^High^) group of cancer patients in the indicated TCGA datasets using Reactome pathway database (positive values in red, negative values in blue). (C) Prognostic value of *CLEC9A* expression for cancer patient overall survival comparing top and bottom quartiles of *sGSN*^Low^ and *sGSN*^High^ subgroups in the indicated TCGA dataset. (D) Comparison between Pearson r correlation values, obtained from correlation of *CLEC9A* or CD8 gene signature with individual MHC class I (cross-)presentation related signature, between *sGSN*^Low^ and *sGSN*^High^ subgroups in the indicated TCGA dataset. (E) Comparison between Pearson r correlation values, obtained from correlation of *CLEC9A*-MHC I antigen processing and presentation signature or *CLEC9A*-ER phagosome pathway signature with individual CD8-MHC class I (cross-)presentation-related signature between *sGSN*^Low^ and *sGSN*^High^ subgroups in the indicated TCGA dataset. (F) Synergistic prognostic value of CD8 and antigen processing and cross-presentation gene signatures comparing quartiles within *sGSN*^Low^ and *sGSN*^High^ subgroups in the indicated TCGA dataset. In (A) data are presented as mean of log2 normalized expression ± SEM survival (Kaplan-Meier) curves in (A, C, and F) were compared using Log-rank (Mantel-Cox) test. Hazard ratios (HR) with 95% confidence interval showed in brackets have been calculated in (A) as a ratio of *sGSN*^Low^ / *sGSN*^High^ group and in (C) as a ratio of each group / *sGSN*^High^*CLEC9A*^Low^. In (B), all the genes were ranked by the Wald’s test and false discovery rate (FDR)-adjusted p values (q values) were calculated. In (D and E) the dotted line indicates a p value of 0.05 obtained by Pearson’s r correlation. ^∗^p ≤ 0.05, ^∗∗^p < 0.01, ^∗∗∗^p < 0.001, ^∗∗∗∗^p < 0.0001; ns, not significant. See also Figure S5 and Table S1.

**Figure S5:**
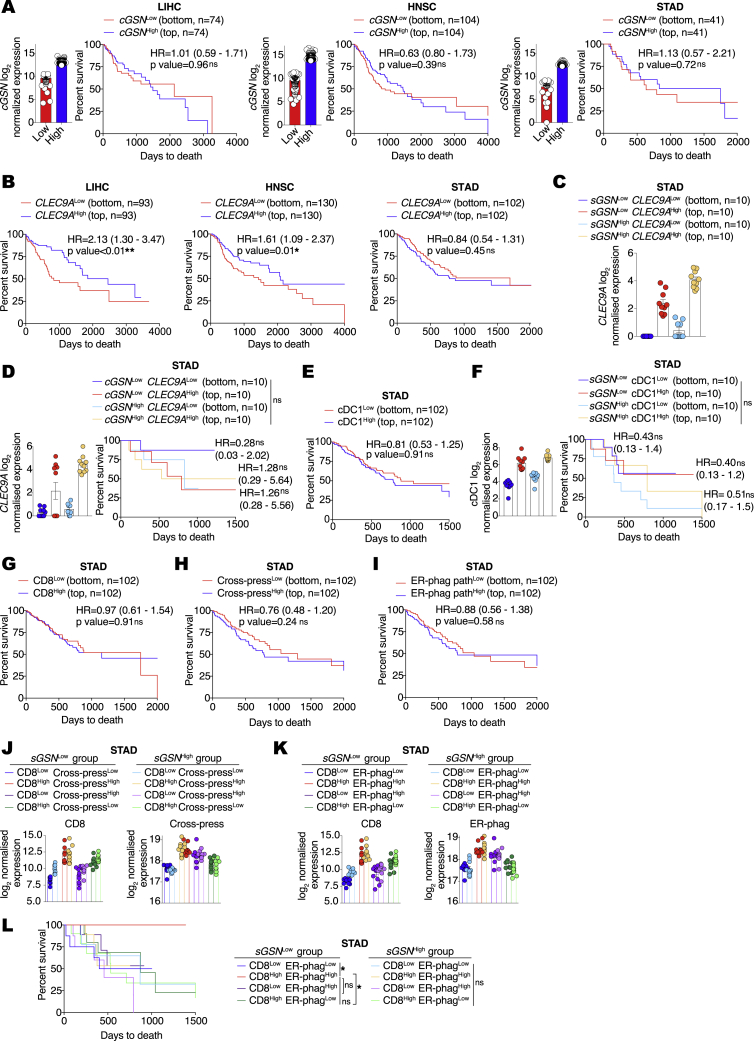
Gene expression in human biopsies of LIHC, HNSC, and STAD tumors and association with patient survival, related to Figure 6 (A) Prognostic value of cytoplasmic gelslolin (*cGSN*) transcript levels for overall survival comparing samples with lowest (*cGSN*^Low^) and highest (*cGSN*^High^) expression in the indicated TCGA datasets. Liver hepatocellular carcinoma (LIH), bottom (n = 74) and top (n = 74) 20% of patient cohort. Head and neck squamous cell carcinoma (HNSC), bottom (n = 104) and top (n = 104) 20% of patient cohort. Stomach adenocarcinoma (STAD), bottom (n = 41) and top (n = 41) 10% of patient cohort. (B) Prognostic value of *CLEC9A* expression for cancer patient overall survival comparing top and bottom quartiles in the indicated TCGA datasets. (C) Transcript levels of *CLEC9A* expression comparing top and bottom quartiles of *sGSN*^Low^ and *sGSN*^High^ subgroups in the indicated TCGA datasets. (D) Prognostic value of *CLEC9A* transcript levels expression for cancer patient overall survival comparing top and bottom quartiles of *cGSN*^Low^ and *cGSN*^High^ subgroups in the indicated TCGA dataset. (E) Prognostic value of cDC1 gene signature expression for cancer patient overall survival comparing top and bottom quartiles in the indicated TCGA dataset. (F) Prognostic value of cDC1 gene signature expression for cancer patient overall survival comparing top and bottom quartiles of *sGSN*^Low^ and *sGSN*^High^ subgroups in the indicated TCGA dataset. (G–I) Prognostic value of (G) CD8 gene signature, (H) antigen processing and cross-presentation gene signature, (I) ER phagosome pathway gene signature expression for cancer patient overall survival comparing top and bottom quartiles in the indicated TCGA datasets. (J) Transcript levels of CD8 and antigen processing and cross-presentation gene signatures comparing quartiles within *sGSN*^Low^ and *sGSN*^High^ subgroups in the indicated TCGA dataset. (K and L) Transcript levels and synergistic prognostic value of CD8 and ER-phagosome pathway gene signatures comparing quartiles within *sGSN*^Low^ and *sGSN* subgroups in the indicated TCGA dataset. In (C, D, F, J, K, L) for *cGSN* and *sGSN* segregation between the highest and lowest expressors the same cut-off was used as in (A) for the indicated TCGA dataset. In (A, C, D, F, J, K) data are presented as mean of log2 normalized expression ± SEM Survival (Kaplan-Meier) curves in (A, B, D-F, G-I, L) were compared using Log-rank (Mantel-Cox) test. Hazard ratios (HR) with 95% confidence interval showed in brackets have been calculated in (A, B, E and G-I) as a ratio of Low expressed transcript /High expressed transcript group, in (D) as a ratio of each group / c*GSN*^High^*CLEC9A*^Low^ and in (F) as a ratio of each group / s*GSN*^High^cDC1^Low^. ^∗^p ≤ 0.05, ^∗∗^p < 0.01, ns, not significant.

Given the results in Figure 3F, we hypothesized that, for some cancers, production of sGSN by tumor (Asare-Werehene et al., 2020; Chen et al., 2017; Tsai et al., 2012) and tumor-infiltrating cells could lead to elevated levels of the protein in the TME irrespective of the amount circulating in plasma, impacting immunity and patient outcome. We performed *in silico* analysis of gelsolin isoform expression using data from The Cancer Genome Atlas (TCGA; https://www.cancer.gov/tcga) for 10 cancers including skin, liver, breast, lung, pancreatic, prostate, low-grade glioma (LGG), head and neck, stomach, and colorectal. Due to the limited dynamic range of *sGSN* transcript levels, slightly different cutoffs were used for different cancers in order to allow for maximum segregation between the highest and lowest expressors while retaining enough data points for comprehensive analysis. For seven cancer types, expression levels of *sGSN* did not impact overall survival irrespective of the cutoff chosen (data not shown). However, analysis of liver hepatocellular carcinoma (LIHC, n = 370), head and neck squamous cell carcinoma (HNSC, n = 518) and stomach adenocarcinoma (STAD, n = 408) revealed that lower *sGSN* transcript expression correlated positively with survival (Figure 6A), a difference that was not attributable to age, sex, or disease stage (Table S1). In the same cancers, the expression of the cytoplasmic gelsolin isoform (*cGSN*) did not correlate with patient survival, highlighting a specific association of sGSN but not cGSN with cancer progression (Figure S5A). Comparison of low versus high *sGSN* tumors using REACTOME pathway analysis revealed that *sGSN*^*Low*^ LIHC, HNSC, and STAD cancers displayed specific enrichment for gene signatures of antigen processing, MHC class I (cross-)presentation, cell death and, except for STAD, gene signatures of adaptive immunity (Figure 6B). Thus, the survival benefit seen in the low *sGSN* group of LIHC, HNSC, and STAD tumors is broadly associated with gene signatures of anti-tumor immunity.

Separately, we determined the prognostic value of *CLEC9A* transcript levels in overall cancer survival by comparing top and bottom patient quartiles (Böttcher et al., 2018). *CLEC9A* expression correlated positively with patient overall survival in LIHC and HNSC but not in the STAD dataset (Figure S5B). The latter therefore allowed us to examine whether *CLEC9A* expression predicted overall survival selectively in the low *sGSN* STAD patient group. Strikingly, we found that this was the case (Figures 6C and S5C) and that it was specific for sGSN, as higher *CLEC9A* expression did not correlate with survival when patients were stratified on the basis of expression of cGSN (Figure S5D). *CLEC9A* is a marker of cDC1s, but a specific cDC1 gene signature (Böttcher et al., 2018) did not associate with STAD patient survival irrespective of *sGSN* expression levels (Figures S5E and S5F), which suggests that the association of *CLEC9A* with patient survival in the low *sGSN* patient group might predominantly reflect DNGR-1 receptor function rather than intratumoral cDC1 abundance. Interestingly, both *CLEC9A* and an “effector CD8 T cell” gene signature (Böttcher et al., 2018) correlated with “MHC class I (cross-)presentation-related” gene signatures more strongly in the low *sGSN* than in the high *sGSN* subgroup of STAD patients (Figure 6D). Furthermore, *CLEC9A* and “effector CD8 T cell” gene signature also cross-correlated to a greater extent in the low *sGSN* subgroup when compared together as part of “MHC class I (cross-)presentation related” gene signatures, highlighting their potential intersection in a common pathway (Figure 6E). Importantly, by examining the top and bottom quartiles as described before (Mariathasan et al., 2018), we found that, although “effector CD8 T cell” and “cross-presentation related” gene signatures did not associate with survival in STAD patients on their own (Figures S5G and S5I), in conjunction, they were able to predict survival selectively in the low *sGSN* patient subgroup, much like *CLEC9A* expression (Figures 6F and S5J–S5L). Thus, in humans, as in mice, *sGSN* expression is associated with poorer cancer outcome, which correlates with lower CLEC9A-CD8 T cell immune-mediated control.

### Low *sGSN* expression in human cancers positively correlates with survival in a subcohort of patients carrying mutations in F-actin-binding proteins

**Figure 7:**
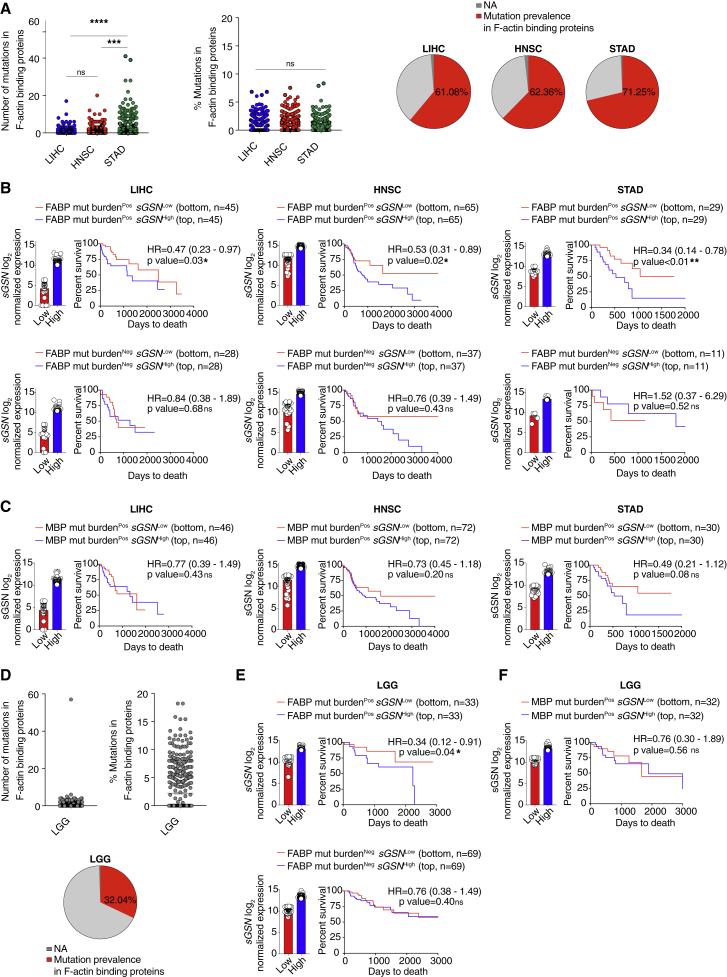
Low *sGSN* levels in human cancer biopsies correlate with patient survival on the basis of mutational prevalence in F-actin-binding proteins (A–F) (A and D) Number (left), frequency (percentage of total mutations, middle), and prevalence (percentage of tumors with ≥1 mutation in the indicated class of genes, right) of mutation in F-actin-binding proteins in the indicated TCGA datasets. (B and E) Prognostic value of *sGSN* transcript levels for overall survival comparing samples with lowest (*sGSN*^Low^) and highest (*sGSN*^High^) expression in the presence (Pos) or absence (Neg) of tumor mutational burden in F-actin-binding proteins (FABPs) in the indicated TCGA datasets. (C and F) Prognostic value of *sGSN* transcript levels for overall survival comparing samples with lowest (*sGSN*^Low^) and highest (*sGSN*^High^) expression in the presence (Pos) or absence (Neg) of mutational burden in microtubule-binding proteins (MBPs) for cancer patient overall survival in the indicated TCGA datasets. For *sGSN* segregation between the highest and lowest expressors the same cutoffs were used as in Figures 6A and S6D for the indicated TCGA datasets. In (A–F) data are presented as mean of counts, frequency or log2 normalized expression ± SEM. In (A and D), all data are presented as mean ± SEM and were analyzed using Dunn’s-corrected Kruskal-Wallis (one-way ANOVA). Survival (Kaplan-Meier) curves in (B, C, E, and F) were compared using Log-rank (Mantel-Cox) test. Hazard ratios (HR) with 95% confidence interval showed in brackets have been calculated in (B, C, E, and F) as a ratio of *sGSN*^Low^ / *sGSN*^High^ group. NA, not applicable. ^∗^q ≤ 0.05, ^∗∗^p < 0.01, ^∗∗∗^p < 0.001, ^∗∗∗∗^p < 0.0001; ns, not significant. See also Figures S6 and S7 and Tables S2 and S3.

**Figure S6:**
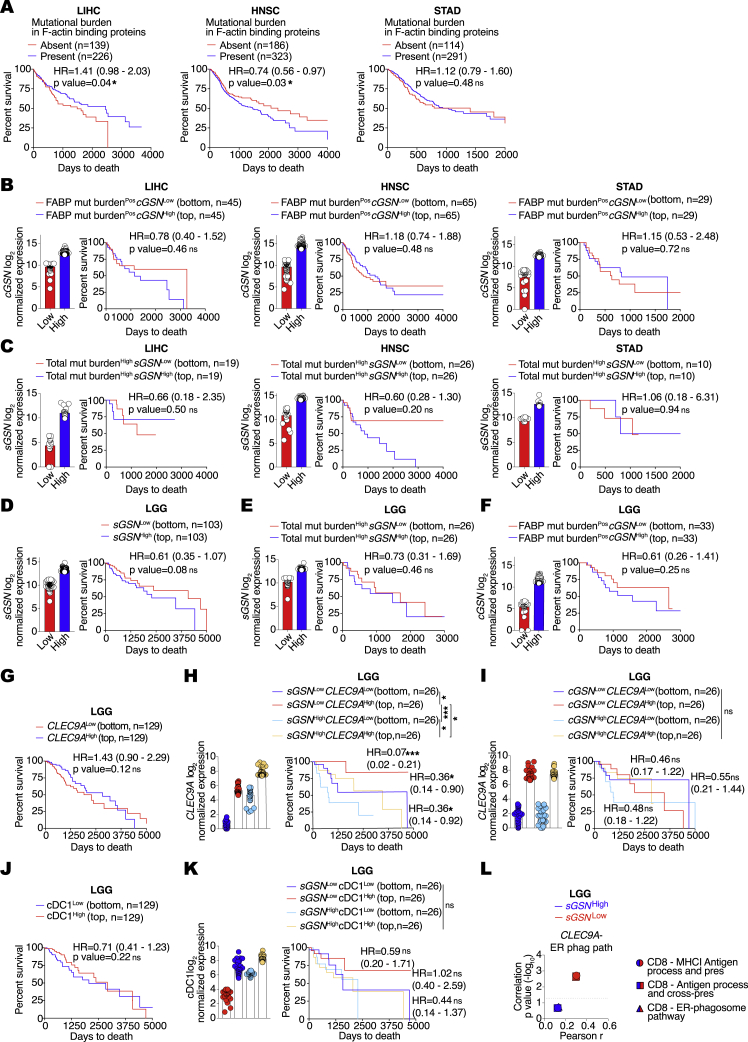
Total and cytoskeleton binding protein-specific tumor mutational burden in human biopsies and their association with patient survival, related to Figure 7 (A) Prognostic value of presence or absence of mutational burden in F-actin binding proteins for cancer patient overall survival in the indicated TCGA datasets. (B) Prognostic value of *cGSN* transcript levels for overall survival comparing samples with lowest (*cGSN*^Low^) and highest (*cGSN*^High^) expression in the presence (Pos) or absence (Neg) of tumor mutational burden in F-actin binding proteins (FABP) in the indicated TCGA datasets. (C) Prognostic value of *sGSN* transcript levels for overall survival comparing samples with lowest (*sGSN*^Low^) and highest (*sGSN*^High^) expression in the high tumor mutational burden (top quartile) patient subcohort of the indicated TCGA datasets. In (B and C) for *cGSN* and *sGSN* segregation between the highest and lowest expressors the same cut-off was used as in (Figure 6A) for the indicated TCGA datasets. (D) Prognostic value of *sGSN* transcript levels for overall survival comparing samples with lowest (*sGSN*^Low^) and highest (*sGSN*^High^) expression in the indicated TCGA dataset. Low grade glioma (LGG), bottom (n = 103) and top (n = 103) 20% of patient cohort. (E) Prognostic value of *sGSN* transcript levels for overall survival comparing samples with lowest (*sGSN*^Low^) and highest (*sGSN*^High^) expression in the high tumor mutational burden (top quartile) patient subcohort of the indicated TCGA dataset. (F) Prognostic value of *cGSN* transcript levels for overall survival comparing samples with lowest (*sGSN*^Low^) and highest (*sGSN*^High^) expression in the presence (Pos) of tumor mutational burden in F-actin binding proteins (FABP) in the indicated TCGA dataset. (G) Prognostic value of *CLEC9A* expression for cancer patient overall survival comparing top and bottom quartiles in the indicated TCGA dataset. (H) Prognostic value of *CLEC9A* expression for cancer patient overall survival comparing top and bottom quartiles of *sGSN*^Low^ and *sGSN*^High^ subgroups in the indicated TCGA dataset. (I) Prognostic value of *CLEC9A* transcript levels expression for cancer patient overall survival comparing top and bottom quartiles of *cGSN*^Low^ and *cGSN*^High^ subgroups in the indicated TCGA dataset. (J) Prognostic value of cDC1 gene signature expression for cancer patient overall survival comparing top and bottom quartiles in the indicated TCGA dataset. (K) Prognostic value of cDC1 gene signature expression for cancer patient overall survival comparing top and bottom quartiles of *sGSN*^Low^ and *sGSN*^High^ subgroups in the indicated TCGA dataset. In (E, F, H, I, K) for *cGSN* and *sGSN* segregation between the highest and lowest expressors the same cut-off was used as in (D) for the indicated TCGA dataset. (L) Comparison between Pearson r correlation values, obtained from correlation of *CLEC9A* – ER phagosome pathway signature with individual CD8 - MHC class I (cross)-presentation related signature between *sGSN*^Low^ and *sGSN*^High^ subgroups in the indicated TCGA dataset. In (B-F, H, I, K) all data are presented as mean of log2 normalized expression ± SEM Hazard ratios (HR) with 95% confidence interval showed in brackets have been calculated in (A-G, J) as a ratio of low expressed transcript or absent mutational burden / high expressed transcript or present mutational group, in (H) as a ratio of each group / s*GSN*^High^*CLEC9A*^Low^, in (I) as a ratio of each group / c*GSN*^High^*CLEC9A*^Low^ and in (K) as a ratio of each group / s*GSN*^High^cDC1^Low^. Survival (Kaplan-Meier) curves in (A-K) were compared using Log-rank (Mantel-Cox) test. In (L) the dotted line indicates a p value of 0.05 obtained by Pearson’s r correlation. ^∗^p ≤ 0.05, ^∗∗∗^p < 0.001. ns, not significant.

**Figure S7:**
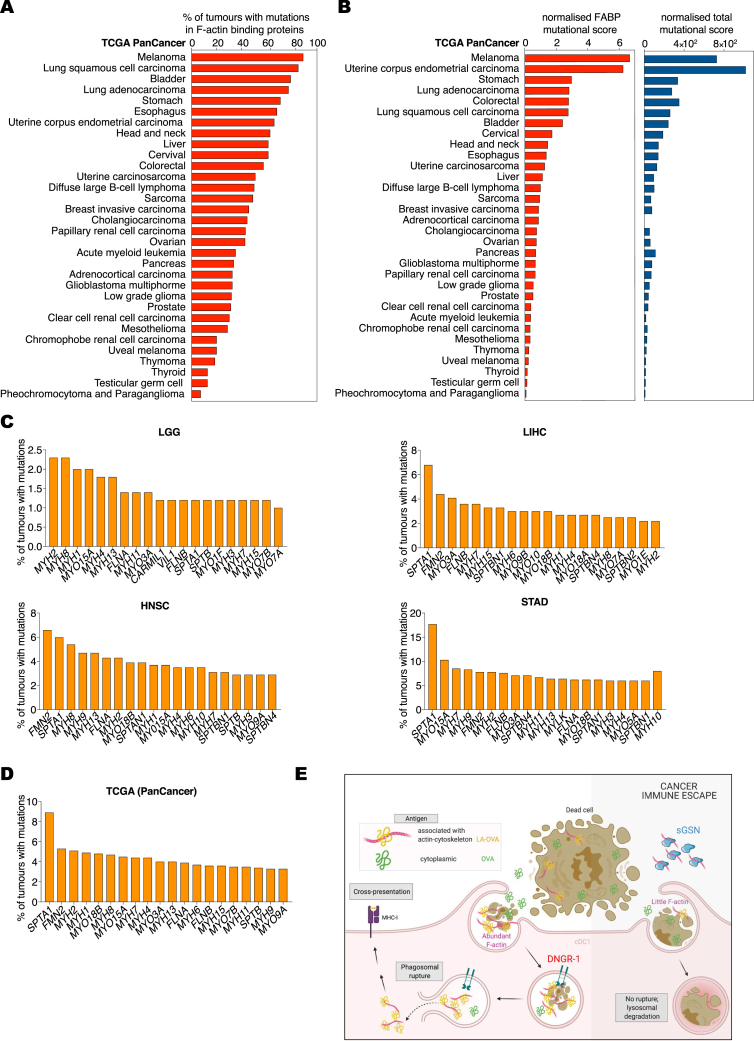
Prevalence of mutations in F-actin-binding proteins in human cancers, related to Figure 7 (A) Mutational prevalence presented as percentage of tumors with ≥ 1 mutation in F-actin binding proteins in the indicated TCGA datasets. (B) Normalized F-actin binding proteins (FABP; left) or total (right) mutational scores are defined as number of mutations per number of tumors in the indicated TCGA datasets. (C and D) Top 20 frequently mutated F-actin binding proteins (C) as percentage of total mutation count of tumors in LGG, LIHC, HNSC, STAD datasets and (D) as percentage of total mutation counts of tumors among all the TCGA datasets listed in (A and B). (E) Schematic summary of the findings: sGSN in the TME promotes cancer immune evasion by inhibiting F-actin binding to DNGR-1, thus, leading to impairement of phagosomal rupture in cDC1 and subsequent cross-presentation preferentially of neoantigens associated with actin cytoskeleton. Image was generated with BioRender.

As described above, DNGR-1-dependent control of cancer in *sGsn*^*−/−*^ mice was most marked for transplantable tumors bearing the LA-OVA model antigen. This suggested that neoantigens resulting from mutations in proteins that associate with F-actin might be preferentially immunogenic in *sGSN*^Low^ patients. We therefore examined LIHC, HNSC, and STAD patients for mutational burden in F-actin-binding proteins (FABPs; Table S2) compared to total mutational burden or, as a specificity control, mutational burden in microtubule-binding proteins (MBPs; Table S3). In LIHC, HNSC, and, in particular, STAD we identified multiple patients with one or more non-silent mutations in the coding regions of one or more genes encoding FABPs (Figure 7A). LIHC but not HNSC and STAD patients bearing FABP mutations displayed better overall survival in the absence of additional stratification (Figure S6A). However, when patients were further stratified by intratumoral *sGSN* transcript levels, it became obvious that the combination of low *sGSN* together with mutations in FABP offered the best correlation with overall survival across all three cancer types (Figure 7B). This was not seen when the analysis was performed using (cytoplasmic) *cGSN* transcripts as the binning criterion (Figure S6B). Further, it was specific to patients with mutations in FABPs as it was not seen when stratification was carried out on the basis of total mutational burden (Figure S6C) or mutations in MBPs (Figure 7C). Moreover, even in a cancer such as LGG (n = 515), in which low *sGSN* expression did not by itself predict survival (Figure S6D), mutational burden in FABPs but not total mutational burden or mutations in MBPs revealed a correlation with intratumoral *sGSN* (Figures 7D–7F and S6E) but not *cGSN* (Figure S6F) expression at the level of overall survival. As seen with STAD patients, increased patient survival in LGG required the intersection of low levels of *sGSN* transcripts and high levels of *CLEC9A* transcripts (Figures S6G and S6H). In contrast, the intersection of *CLEC9A* expression with expression of cytoplasmic *cGSN* did not correlate with survival (Figure S6I), and a cDC1 gene signature did not substitute for *CLEC9A* (Figures S6J and S6K). Furthermore, as observed with STAD patients, in the sub-group of LGG with lower *sGSN* expression, there was a strong correlation between gene signatures for “*CLEC9A*-ER phagosome pathway” and “effector CD8 T cell-cross-presentation related” (Figure S6L). Collectively, these data suggest that low sGSN expression may selectively enhance immune responses to neoantigens associated with the actin cytoskeleton and patient survival even in cancers such as LGG and LIHC with low mutational burden (Figures S7A–S7D).

## Discussion

cDC1s have emerged as key players in cross-priming of anti-tumor CD8^+^ T cells and in the response to cancer immunotherapy (Broz et al., 2014; Hildner et al., 2008; Salmon et al., 2016; Sánchez-Paulete et al., 2016; Spranger et al., 2017). We and others have shown that cancer cells can block recruitment of cDC1s to escape CD8^+^ T cell immunosurveillance (Böttcher et al., 2018; Spranger et al., 2015; Zelenay et al., 2015). However, little is known as to whether antigen acquisition and presentation functions of cDC1s are impacted by tumor- or host-derived factors. Furthermore, although the abundance of the cDC1 hallmark transcripts, such as *CLEC9A*, in tumors correlates positively with cancer patient survival (Barry et al., 2018; Böttcher et al., 2018; Broz et al., 2014; Michea et al., 2018), it is unclear if this reflects a role of the corresponding gene products in cDC1-mediated anti-tumor immunity. Here, we show that the *CLEC9A* product, DNGR-1, can promote cross-presentation of dead tumor cell antigens leading to anti-tumor immunity but that this effect is often masked by sGSN produced either locally in the TME or circulating in plasma. Thus, sGSN can dictate the degree to which tumor antigenicity is revealed to the CD8^+^ T cell compartment by cDC1s via DNGR-1-mediated cross-presentation.

DNGR-1 functions in cDC1s by recognizing F-actin on necrotic cargo and signaling to promote phagosomal rupture, with consequent access of dead cell-associated antigens to the cytosolic MHC class I antigen processing pathway (Canton et al., 2021). Rupture is determined in part by the extent of DNGR-1 triggering and therefore is expected to be biased toward phagosomes containing debris with the highest actin cytoskeletal content. Together with the fact that the debris is at various stages of dissolution and leaching its soluble contents, it is likely that DNGR-1-dependent phagosomal rupture will bias cross-presentation toward those antigens that are most intimately associated with actin filaments (Figure S7E). Indeed, our findings suggest that DNGR-1-dependent cross-presentation, when not blocked by sGSN, favors cross-priming of anti-tumor CD8^+^ T cells specific for mutated proteins that are part of the actin cytoskeleton (Figure S7E). Interestingly, mutations in FABPs occur frequently in the vast majority of human cancers and can generate tumor neoantigens in both mice and humans (Matsushita et al., 2012; Zorn and Hercend, 1999). The fact that such mutations correlate with better prognosis specifically in patients whose tumors have relatively low *sGSN* transcript levels suggests that local production of sGSN in the TME may be a means of evading DNGR-1-dependent induction of anti-tumor immunity irrespective of plasma sGSN levels. Consistent with that notion, we show that ectopic expression of sGSN is sufficient to promote immune escape of murine tumors even in *sGsn*^*−/−*^ mice (Figure 3F).

In our analysis, the prognostic value of TME *sGSN* expression was restricted to specific cancer subtypes (liver, head and neck, stomach cancers, and LGG). The lack of prognostic value of tumor-derived *sGSN* transcript expression in the other six analyzed TCGA datasets could reflect the shortcomings of estimating local sGSN protein levels based on mRNA counts. Consistent with that possibility, high levels of sGSN proteins have been reported in ovarian and prostate cancer and found to be associated with poorer clinical outcome (Asare-Werehene et al., 2020; Chen et al., 2017). However, it is also likely that, for many cancers, local production of sGSN is functionally redundant in the face of high circulating levels of sGSN in plasma. Indeed, our mouse data indicate that plasma sGSN is amply sufficient to dampen anti-tumor immunity in many instances. Interestingly, perhaps related to our findings with *sGsn*-deficient mice, it has been reported that patients with Meretoja’s disease, in which proteolytic cleavage leads to loss of sGSN function, display a lower prevalence of fatal cancers (Schmidt et al., 2016). Finally, irrespective of sGSN, it is important to note that cDC1s and/or DNGR-1-mediated cross-presentation might be dispensable for immunity to some cancer types (Asano et al., 2011; Gilfillan et al., 2018; Ma et al., 2013).

Collectively, our data identify sGSN as a hitherto unsuspected player in tumor evasion of immunity. They further reveal that DNGR-1-mediated cross-presentation favors immune visibility of cancer clones bearing sequence mutations in proteins of the actin cytoskeleton, which are surprisingly common even in patients with low overall mutational burden (Figures S7A and S7B). In physiology, sGSN inhibition of DNGR-1 activity might help prevent inappropriate immune responses to cytoskeletal antigens (e.g., myositis). This remains to be assessed, as does the more general point of the extent to which sGSN acts as a barrier to autoimmunity, which can be explored in the future by testing *sGsn*^*−/−*^ mice. However, we note that these mice, at least on a C57BL/6 background, do not display signs of spontaneous autoimmunity. Transiently targeting the interaction between sGSN and F-actin might therefore constitute a safe and attractive strategy for tumor immunotherapy. If one can circumvent the difficulty posed by the high concentration of sGSN in plasma, sGSN blockade in the TME could boost the antigenic visibility of tumor cells and, in conjunction with checkpoint blockade immunotherapy, help augment cancer control by CD8^+^ T cells.

### Limitations of study

Our results argue that DNGR-1 preferentially promotes cross-presentation of tumor antigens associated with the actin cytoskeleton and that this is opposed by sGSN from circulation or produced by tumor cells. We used LA-OVA, in which OVA is fused to the Lifeact F-actin binding peptide in proof-of-principle studies in mice, but it will be important to extend those findings to tumor cells expressing a bona fide FABP neoantigen. In addition, the full extent to which the DNGR-1-sGSN cross-presentation axis shapes the antigenic repertoire of cancer cells will require analysis of immunoediting of chemically induced cancers (Matsushita et al., 2012) in the different mouse strains described in this report.

## STAR★Methods

### Key resources table

**Table:** 

REAGENT or RESOURCE	SOURCE	IDENTIFIER
**Antibodies**		

Mouse anti-Actin (AC-40)	Sigma	Cat#A4700; RRID:AB_476730
Mouse anti-human Gelsolin AF488 (20)	Novus Biologicals/BioTechne	NBP1-05161AF488
Rat anti-mouse DNGR-1 (1F6)	The Francis Crick Institute	N/A
Rat IgG2a mouse (R19-15)	BD Biosciences	Cat# 562028; RRID:AB_10895561
Mouse anti-mouse/rat XCR-1 BV421 (ZET)	Biolegend	Cat# 148216; RRID:AB_2565230)
Mouse anti-mouse/rat XCR-1 BV785 (ZET)	Biolegend	Cat# 148225; RRID:AB_2783119
Rat anti-mouse CD45 V500 (30-F11)	BD Biosciences	Cat# 561487; RRID:AB_10697046
Mouse anti-mouse CD45.2 BV605 (104)	Biolegend	Cat# 109841; RRID:AB_2563485
Mouse anti-mouse CD45.2 BV711 (104)	Biolegend	Cat# 109847; RRID:AB_2616859
Mouse anti-mouse CD45.2 PerCP/Cy5.5 (104)	BD Biosciences	Cat# 109827; RRID:AB_893352
Rat anti-mouse Ly-6C BV605 (HK1.4)	Biolegend	Cat# 128036; RRID:AB_2562353
Rat anti-mouse Ly-6G FITC (1A8)	Biolegend	Cat# 127605; RRID:AB_1236488
Rat anti-mouse Ly-6G/Ly-6C (Gr-1) PerCP/Cy5.5 (RB6-8C5)	Biolegend	Cat# 108428; RRID:AB_893558
Rat anti-mouse CD8α BV605 (53-6.7)	Biolegend	Cat# 100744; RRID:AB_2562609
Rat anti-mouse CD8α BV421 (53-6.7)	Biolegend	Cat# 100753; RRID:AB_2562558
Rat anti-mouse CD8α APC (53-6.7)	BD Biosciences	Cat# 553035; RRID:AB_398527
Rat anti-mouse CD8α APC/Cy7 (53-6.7)	Biolegend	Cat# 100713; RRID:AB_312752
Rat anti-mouse CD8α FITC (53-6.7)	BD Biosciences	Cat# 553031; RRID:AB_394569
Rat anti-mouse CD45R/B220 BV650 (RA3-6B2)	Biolegend	Cat# 103241; RRID:AB_11204069
Rat anti-mouse CD45R/B220 PerCP/Cy5.5 (RA3-6B2)	Biolegend	Cat# 103236; RRID:AB_893354
Rat anti-mouse/human CD11b FITC (M1/70)	BD Biosciences	Cat# 553310; RRID:AB_394774
Rat anti-mouse CD11b BV650 (M1/70)	BD Biosciences	Cat# 563402; RRID:AB_2738184
Rat anti-mouse CD4 PerCP/Cy5.5 (RM4-5)	BD Biosciences	Cat# 553052; RRID:AB_394587
Rat anti-mouse CD4 PE (RM4-5)	BD Biosciences	Cat# 553049; RRID:AB_394585
Armenian hamster anti-mouse CD103 PerCP/Cy5.5 (2E7)	Biolegend	Cat# 121416; RRID:AB_2128621
Rat anti-mouse CD103 APC (M290)	BD Biosciences	Cat# 562772; RRID:AB_2737784)
Mouse anti-mouse NK1.1 PE (PK136)	BD Biosciences	Cat# 553165; RRID:AB_394677
Mouse anti-mouse NK1.1 FITC (PK136)	Biolegend	Cat# 108706; RRID:AB_313393
Armenian hamster anti-mouse TCR γδ PE/Cy7 (GL3)	Biolegend	Cat# 118124; RRID:AB_11204423
Mouse anti-mouse CD64 PE/Cy7 (X54-5/7.1)	Biolegend	Cat# 139314; RRID:AB_2563904
Mouse anti-mouse CD64 BV421 (X54-5/7.1)	BD Biosciences	Cat# 740622; RRID:AB_2740319
Rat anti-mouse Sirpα (CD172α) AF647 (P84)	Biolegend	Cat# 144028; RRID:AB_27;1301
Rat anti-mouse Sirpα (CD172α) APC/Fire 750 (P84)	Biolegend	Cat# 144030; RRID:AB_2721317
Armenian hamster anti-mouse CD3e APC (145-2C11)	BD Biosciences	Cat# 553066; RRID:AB_398529
Armenian hamster anti-mouse CD3e APC-eFluor 780 (145-2C11)	E-Bioscience	Cat# 47-0031-82; RRID:AB_11149861
Rat anti-mouse MHC-II (I-A/I-E) AF700 (M5/114.15.2)	E-Bioscience	Cat# 56-5321-82; RRID:AB_494009
Rat anti-mouse MHC-II (I-A/I-E) FITC (M5/114.15.2)	E-Bioscience	Cat# 11-5321-85; RRID:AB_465233
Armenian hamster anti-mouse CD11c APCeFluor780 (N418)	E-Bioscience	Cat# 47-0114-82; RRID:AB_1548652
Armenian hamster anti-mouse CD11c BV421 (N418)	Biolegend	Cat# 117329; RRID:AB_10897814
Armenian hamster anti-mouse TCRβ APC/Cy7 (H57-597)	Biolegend	Cat# 109220; RRID:AB_893624
Rat anti-mouse F4/80 AF647 (BM8)	Thermo Fisher Scientific	Cat# MF48021; RRID:AB_10375289
Rat anti-mouse Siglec F PE (E50-2440**)**	BD Biosciences	Cat#55212; RRID:AB_394341
Rat anti-mouse CD62L FITC (MEL-14)	BD Biosciences	Cat# 553150; RRID:AB_394665
Rat anti-mouse CD44 APC-eFluor 780 (IM7)	E-Bioscience	Cat# 47-0441-82; RRID:AB_1272244
Rat anti-mouse CD44 APC (IM7)	BD Biosciences	Cat# 559250; RRID:AB_398661
Rat anti-mouse CD206 BV421 (C068C2)	Biolegend	Cat# 141717; RRID:AB_2562232
Rat anti-mouse CD86 BV711 (GL-1)	BD Biosciences	Cat# 740688; RRID:AB_2734766
Rat anti-mouse CD19 BV421 (6D5)	Biolegend	Cat# 115538; RRID:AB_11203527
Rat anti-mouse CD19 AF700 (6D5)	Biolegend	Cat# 115528; RRID:AB_49373
Mouse anti-mouse GATA-3 BV421 (16E10A23)	Biolegend	Cat# 653814; RRID:AB_2563221
Mouse anti-mouse RORγt BV650 (Q31-378)	BD Biosciences	Cat# 564722; RRID:AB_2738915
Rat anti-mouse FOXP3 PE (FJK-16 s)	E-Bioscience	Cat# 12-5773-82; RRID:AB_465936
Mouse anti-mouse T-bet APC (4B10)	BioLegend	Cat# 644814; RRID:AB_10901173
Rat anti-mouse CD16/CD32 (2.4G2)	BD Biosciences	Cat# 553141; RRID:AB_394656
InVivoMAb rat anti-mouse PD-1 (CD279) (RMP1-14)	Bio X Cell	Cat# BE0146; RRID:AB_10949053
InVivoMAb rat IgG2a isotype control (2A3)	Bio X Cell	Cat# BE0089; RRID:AB_1107769
InVivoPlus mouse anti-mouse CTLA-4 (CD152) (9D9)	Bio X Cell	Cat# BE0164; RRID:AB_10949609
InVivoMAb mouse IgG2b isotype control (MPC-11)	Bio X Cell	Cat# BE0086; RRID:AB_1107791
InVivoMAb rat anti-mouse CD8α (2.43)	Bio X Cell	Cat# BE0061; RRID:AB_1125541
InVivoMAb rat IgG2b isotype control	Bio X Cell	Cat# BE0090; RRID:AB_1107780
Mouse anti-mouse DNGR-1 (7H11)	The Francis Crick Institute	N/A
Rat anti-mouse IFN-γ ELISA capture (R4-6A2)	BD Biosciences	Cat# 551216; RRID:AB_394094
Rat anti-mouse IFN-γ ELISA detection (XMG1.2)	BD Biosciences	Cat# 554410; RRID:AB_395374
Goat anti-mouse IgG Biotin ELISA detection	SouthernBiotech	Cat# 1030-08 RRID: AB_2794296
Mouse anti-FLAG-HRP (M2)	Sigma-Aldrich	Cat# A8592; RRID:AB_439702
Rabbit anti-mouse Gelsolin (D9W8Y)	Cell Signaling Technology	Cat# 12953; RRID:AB_2632961
Mouse anti-mouse β-Actin-HRP (AC-15)	Sigma-Aldrich	Cat# A3854; RRID:AB_262011
Rabbit anti-Ovalbumin (OVA; Egg-White) polyclonal	Sigma-Aldrich	ABS818
Goat anti-rabbit IgG(H+L), mouse/human-HRP polyclonal	SouthernBiotech	Cat# 4050-05; RRID:AB_2795955
Goat anti-mouse IgG (H+L)-HRP polyclonal	Thermo Fisher Scientific	Cat# G-21040; RRID:AB_2536527
Goat anti-mouse IgG (H+L) AF488 polyclonal	Thermo Fisher Scientific	Cat# A28175 RRID: AB_2536161

**Bacterial and virus strains**		

pMSCV-IRES-OVA-mCherry (retrovirus pseudotype)	This paper	N/A
pMSCV-IRES-Life-Act-OVA-mCherry (retrovirus pseudotype)	This paper	N/A
PLKO.1-puro-Gsn^shRNA^ (lentivirus)	This paper	N/A
Influenza A virus (X31)	The Francis Crick Institute	N/A

**Chemicals, peptides, and recombinant proteins**		

Collagenase IV	Worthington	LS004188
DNASE I	Roche	11284932001
LIVE/DEAD Fixable Blue Dead Cell Stain Kit	Life Technologies	L34962
LIVE/DEAD® Fixable Aqua Dead Cell Stain Kit	Life Technologies	L34957
Fixation Medium A	Nordic MUbio	GAS-002A-1
CPRG Chlorophenol red-β-D-galactopyranoside	Roche	10884308001
Poly(I:C) (HMW) VacciGrade	Invivogen	Vac-pic
R-PE-conjugated H-2K^b^ /SIINFEKL pentamer	Proimmune	F093-2C-G
R-PE-conjugated H-2D^b^/ASNENMETM Influenza A NP 366-374 Pentamer	Proimmune	F119-2A-G
Albumin from chicken egg white (OVA)	Sigma	A5503
Albumin prepared from chicken eggs	Boes et al., 2003	N/A
OVA peptide (SIINFEKL)	The Francis Crick Institute	N/A
ExtrAvidin-Alkaline Phosphatase	Sigma	E2636
SIGMAFAST p-nitrophenyl phosphatase tablets	Sigma	N2770-50SET
Amersham Protran nitrocellulose blotting membrane	Cytiva	10600001
Recombinant human plasma Gelsolin	Cytoskeleton Inc.	HPG6-A
Recombinant human Cofilin 1	Cytoskeleton Inc.	CF01-A
Actin from skeletal muscle	Cytoskeleton Inc.	AKL99
Actin biotin-conjugated	Cytoskeleton Inc	AB07
Actin rhodamine-conjugated	Cytoskeleton Inc	AR05
Myosin II from rabbit skeletal muscle	Cytoskeleton Inc.	MY02
Actin Polymerization buffer (10x)	Cytoskeleton Inc	BSA02-001
Flag-tagged dimeric mDNGR-1 ECD	Ahrens et al., 2012	N/A

**Critical commercial assays**		

TissueLyser II	QIAGEN	https://www.qiagen.com/us/products/human-id-and-forensics/automation/tissuelyser-ii/
QiaShredder	QIAGEN	https://www.qiagen.com/gb/products/instruments-and-automation/accessories/qiashredder/#orderinginformation
RNeasy Mini Kit	QIAGEN	https://www.qiagen.com/gb/products/discovery-and-translational-research/dna-rna-purification/rna-purification/total-rna/rneasy-mini-kit/#orderinginformation
SuperScritpt II Reverse Transcriptase	Thermo Fisher Scientific	18064022
PowerUp SYBR Green Master Mix	Thermo Fisher Scientific	A25741
Foxp3 / Transcription Factor Staining Buffer Set	E-Bioscience	00-5523-00
EasySep Mouse Naive CD8^+^ T Cell Isolation Kit	STEMCELL Technologies	19858
Cytometric bead array (CBA)	BD Biosciences	https://www.bdbiosciences.com/us/reagents/research/immunoassays/cytometric-bead-array/bd-cytometric-bead-array-cba-kits/c/745097

**Deposited data**		

Genotype-Tissue Expression (GTEx)	The Broad Institute	https://gtexportal.org
The Cancer Genome Atlas (TCGA)	Firehose, The Broad Institute	https://gdac.broadinstitute.org/
REACTOME pathway database	(Jassal et al., 2020)	https://reactome.org

**Experimental models: cell lines**		

bm1OVAMEF	C. Reis e Sousa (Sancho et al., 2009)	N/A
BWZ	C. Reis e Sousa (Sancho et al., 2009)	N/A
MutuDC1940	(Fuertes Marraco et al., 2012)	N/A
5555 Braf^V600E^	C. Reis e Sousa (Zelenay et al., 2015)	N/A
MCA-205	George Kassiotis	N/A
EG-7	The Francis Crick Institute	N/A
B16F10 OVA-GFP	The Francis Crick Institute	N/A
5555 Braf^V600E^*Gsn* KD	This paper	N/A
B16F10 LA-OVA-mCherry	This paper	N/A
MCA-205 OVA-mCherry	This paper	N/A
MCA-205 LA-OVA-mCherry	This paper	N/A
MCA-205 LA-OVA-mCherry-cGSN	This paper	N/A
MCA-205 LA-OVA-mCherry-sGSN	This paper	N/A

**Experimental models: organisms/strains**		

C57BL/6J (WT)	The Francis Crick Institute	N/A
*sGsn*^*−/−*^ (C57BL/6-*Gsn*^*em2(sGsn)Crs*^)	This paper	N/A
*Clec9a*^*gfp/gfp*^ (B6(Cg)-*Clec9a*^*tm1.1Crs*^)	(Sancho et al., 2009)	N/A
*Clec9a*^*cre/cre*^ (B6J.B6N(Cg)-*Clec9a*^*tm2.1(icre)Crs*^)	(Schraml et al., 2013)	N/A
*sGsn*^*−/−*^*;Clec9a*^*gfp/*^*gfp (C57BL/6-Gs*^*nem2(sGsn)Crs*^*; Clec9a*^*tm1.1Crs*^*)*	This paper	N/A
*sGsn*^*−/−*^*;Gc*^*−/−*^*(C57BL/6-Gsn*^*em2(sGsn)Crs*^*; Gc*^*tm1.1(KOMP)Vlcg*^*)*	This paper	N/A
*OT-I x Rag1*^*−/−*^*(B6.129-Tg(TcraTcrb)1100Mjb ; Rag1*^*tm1Bal*^*)*	The Francis Crick Institute	N/A
*N. brasiliensis*	Judy Allen	N/A

**Oligonucleotides**		

Silencing-Mouse *Gsn*-shRNA-antisense: TTCAGACACGTGTACTTGAGC	Dharmacon Horizon Discovery	TRCN0000071930
Cloning-Primer *cGsn/sGsn* -Forward: CCCCAAGCTTGGCCTTCAGGCA GCCAGCTCAGC	This paper	N/A
Cloning Primer *cGsn* - Reverse: ACCC CAAGCTGGCCTCTGAGGCCATGG TGGTGGAGCACCCC	This paper	N/A
Cloning-Primer *sGsn* -Reverse: ACCC CAAGCTGGCCTCTGAGGCCA TGGCTCCGTACCGCTCTTC	This paper	N/A

**Recombinant DNA**		

pVSV-G	C.Reis e Sousa	N/A
pHIV (gag-pol)	C.Reis e Sousa	N/A
pSBbi-GFP-hygromycin resistant vector	Addgene	605414
pCMV(CAT)T7-SB100 (SB100X transposase)	Addgene	34879

**Software and algorithms**		

GraphPad Prism v7	GraphPad	https://www.graphpad.com/scientific-software/prism/
FlowJo v10.7.1	FlowJo	https://www.flowjo.com
cBioportal	TCGA Pan-Cancer Atlas	https://www.cbioportal.org
R: The Project for Statististical Computing	R project	N/A

### Resource availabilty

#### Lead contact

Further information and requests for resources and reagents should be directed to and will be fulfilled by the lead contact, Caetano Reis e Sousa (caetano@crick.ac.uk).

#### Materials availability

All plasmids, mouse and tumor cell lines generated in this study are available from the lead contact.

#### Data and code availability

This study did not generate datasets/code.

### Experimental model and subject details

#### Mice

Mice selectively lacking sGSN (*sGsn*^*−/−*^) were generated by microinjection of mRNA Cas9(D10A) and *in vitro* transcribed paired guide RNAs (gRNAs), targeting the alternatively-spliced exon coding for the signal peptide of the sGSN gene product, into fertilized single cell staged C57BL/6J embryos (Figure 1F). Embryos carrying correctly targeted mutations were selected and founder lines were established. One founder line carrying the targeted allele *Gsn*^*em2(sGsn)Crs*^ (Figure 1G) was designated *sGsn*^*−/−*^ and used for these studies. *Gc*^*−/−*^ mice carrying the *Gc*^*tm1.1(KOMP)Vlcg*^ allele on a C57BL/6 background were purchased from KOMP repository. Mice doubly deficient for either sGSN and DNGR-1 (*sGsn*^*−/−*^*;Clec9a*^*gfp/gfp*^) or sGSN and Gc (*sGsn*^*−/−*^*;Gc*^*−/−*^) were generated by crossing *sGsn*^*−/−*^ mice with either DNGR-1-deficient mice (*Clec9a*^*tm1.1Crs*^ a.k.a., *Clec9a*^*gfp/gfp*^ (Sancho et al., 2009) or *Gc*^*−/−*^ mice (all on a C57BL/6 background). The above mice, as well as C57BL/6, *Clec9a*^*gfp/gfp*^, another line of DNGR-1 deficient mice (*Clec9a*^*cre/cre*^*;* (Schraml et al., 2013) and OT-I x *Rag1*^*−/−*^ mice were bred at the animal facility of the Francis Crick Institute. Mouse genotypes were determined using real time PCR with specific probes designed for each gene (Transnetyx, Cordova, TN). Serum was collected from aged C57BL/6J and *sGsn*^*−/−*^ mice, and sent to the UT Southwestern Medical Centre Microarray Core facility for autoantibody determination using their autoantigen microarray.

Mice were used at 5 – 12 weeks of age for experiments. For tumor challenge, males and females were used as we did not observe sexual dimorphism (not shown). However, in any one experiment, mice were sex-matched and randomly assigned to treatment or control groups. Mice of different genotypes were littermates and/or co-housed for a minimum of 3 weeks before experiments. Animal experiments were performed in accordance with national and institutional guidelines for animal care and were approved by the Francis Crick Institute Biological Resources Facility Strategic Oversight Committee (incorporating the Animal Welfare and Ethical Review Body) and by the Home Office, UK.

#### Cells

The MutuDC1940 line (Fuertes Marraco et al., 2012) was a kind gift from Hans Acha-Orbea and was cultured in IMDM medium containing 10% heat-inactivated FCS, 50 μM 2-mercaptoethanol, 100 units/mL penicillin, 100 μg/mL streptomycin. All other cell lines were grown in RPMI 1640 containing 10% FCS, 2 mM glutamine, 50 μM 2-mercaptoethanol, 100 units/mL penicillin, 100 μg/mL streptomycin (R10). All media and media supplements were from Life Technologies except for FCS (Source Bioscience).

BWZ cells are stably transduced with mouse CLEC9A fused with the ζ-chain of the T cell receptor and express a β-gal reporter for nuclear factor of activated T cells (NFAT) (Sancho et al., 2009). For retroviral transduction of cancer cell lines, retrovirus was packaged in 293T cells transfected with a mixture of plasmids: 2 μg of pVSV-G envelope protein-coding plasmid, 3.72 μg of pHIV (gag-pol) packaging plasmid and 10 μg of pMSCV-IRES-Life-Act-OVA-mCherry plasmid using Lipofectamine 2000 (Invitrogen). After two days post-transfection, the pseudotyped virus-containing culture media was harvested, filtered and used to infect target cells (B16F10 and MCA-205) in the presence of 10 μg/mL polybrene. After two rounds of infection the medium in the target cells was exchanged for fresh complete RPMI1640 medium. For positive clone selection the medium was supplemented with puromycin (1.5 μg/mL for B16F10 and 5 μg/mL for MCA-205) and after three passages target cells were FACS-sorted based on mCherry expression. For lentiviral transduction, 293T cells were co-transfected with a mixture of 2 μg of pVSV-G envelope protein-coding plasmid, 3.72 μg of psPAX2 packaging plasmid and 10 μg of PLKO.1-puro-Gsn^ShRNA^ (mouse shRNA, TRCN0000071930, mature sequence anti-sense: TTCAGACACGTGTACTTGAGC) using Lipofectamine 2000 (Invitrogen). Viral infection and subsequent selection was performed as above. 5555 Braf^V600E^ *Gsn* knockdown (KD) cells were positively selected using puromycin (1 μg/mL) containing medium. The MCA-205 LA-OVA-mCherry expressing either cGSN or sGSN achieved using the sleeping beauty transposon system. In brief, cGSN and sGSN RNA was extracted from mouse muscles, converted into cDNA and subsequently cloned into the pSBbi-GFP-hygromycin (GH) resistant vector (Addgene). MCA-205 LA-OVA-mCherry cells were transfected with a mixture of plasmids: 0.4 ug transposase (Addgene) and 1.6 ug pSBbi-GFP-GH using Lipofectamine 2000 (Invitrogen). For positive clone selection GFP^+^ cells have been sorted using FACS.

### Method details

#### Tumor cell injections

Tumor cells were dissociated with trypsin (0.25%), and washed three times in PBS. The final cell pellet was resuspended and diluted in endotoxin-free PBS (between 0.2 × 10^6^ to 0.5 × 10^6^ cells per 100 μl) and injected s.c. in the shaved right flank of each recipient mouse. Tumor growth was monitored every 1 to 3 days, and the longest tumor diameter (*l*) and perpendicular width (*w*) were measured using digital Vernier callipers; tumor volume was calculated using the formula: *length* × *width*^*2*^ */2* and expressed as mm^3^ (Faustino-Rocha et al., 2013).

##### *In vivo* administration of immune-checkpoint blockade therapy

For immune-checkpoint therapy *in vivo*, anti-PD1 monoclonal antibody (clone RMP1-14, BioXCell, BE0146) or rat IgG2a isotype control (clone 2A3, BioXCell, BE0089) was administered i.p. at 200 μg /200 μL PBS per mouse from day 3 post-tumor cell transplantation, every 3 days up to a maximum of six doses. For the combination therapy of poly(I:C) with anti-CTLA-4, mice received 50 μg / 50 μL of poly(I:C) (VacciGrade, InvivoGen, vac-pic) or 50 μL of PBS injected intratumorally on days 7 and 11 post-tumor cell transplantation, and either anti-CTLA-4 monoclonal antibody (clone 9D9, BioXCell, BP0164) or rat IgG2b isotype control (clone MPC-11, BioXCell BE0086) 50 μg / 200 μL i.p. on days 6 and 12.

##### *In vivo* CD8 T cell depletion

For CD8^+^ T cell depletion, mice received 300 μg / 200 μL of anti-CD8 (clone 2.43, BioXCell, BE0061) or rat IgG2b isotype control (clone LTF-2, BioXCell, BE0090) i.p. from 3 days prior to inoculation of tumor cells and followed twice per week until the end of the experiment (days: 1, 4, 7, 10, 13).

#### Dot blot binding assay and western blot

Binding of DNGR-1 to *in vitro* polymerized F-actin was analyzed by dot blot as described previously (Ahrens et al., 2012; Schulz et al., 2018). Briefly, F-actin was transferred onto nitrocellulose membranes by gravity flow using a dot blot apparatus. Post-transfer, membranes were blocked in 5% milk, cut into strips, and either probed directly as per the published protocol or incubated with mouse serum, heat-inactivated FCS or the purified ABPs in blocking solution (5% milk) for 1-2 h, washed and then probed with FLAG-tagged mDNGR-1 ECD followed by HRP-conjugated mouse anti-FLAG antibody (M2, Sigma, 1:20000 dilution). For western blot of mouse serum, equivalent volumes of serum samples were diluted in Laemmli buffer, resolved using reducing SDS-PAGE and transferred to nitrocellulose membranes (Merck-Millipore). For cytoplasmic gelsolin, splenic lysates were prepared by homogenization using a TissueLyser II (QIAGEN) in cold protein lysis buffer (RIPA supplemented with protease inhibitors (Roche) before quantification of supernatants using BCA (Thermo Fisher Scientific). Equal amounts of protein were diluted in Laemmli buffer, resolved using reducing SDS-PAGE and transferred to nitrocellulose membranes (Merck-Millipore). Secreted, cytoplasmic gelsolin and OVA levels were assessed by probing membranes with anti-gelsolin antibody (D9W8Y, Cell Signaling Technology, 1:1000 dilution) or anti-OVA antibody (polyclonal antibody, Sigma, 1:1000), respectively, followed by HRP-anti-rabbit antibody (1:5000 dilution). Loading controls were developed using HRP-anti-mouse IgG (polyclonal, Life Technologies, 1:10000) for serum or HRP-anti-β-actin (AC-15, Sigma, 1:10000) for lysates. Visualization was carried out with the SuperSignal West Pico Chemiluminescent substrate kit (Thermo Fisher Scientific).

#### Preparation of F-actin

F-actin was prepared as described (Ahrens et al., 2012; Schulz et al., 2018). Briefly, G-actin (10 mg/mL, 200 μM) stock was diluted 1:10 in a mixture of 1x G-actin buffer and 10x F-actin buffer and left at RT for at least 1 h to induce filament formation. Soluble F-actin (20 μM) was then diluted 1:4 in PBS. F-actin was incubated for 1 h at RT and adjusted to the final assay concentration (top dose) with PBS. Dilution series of F-actin preparations were prepared in PBS and used directly for dot blot and reporter cell assays. For coupling to beads, biotinylated, fluorescent F-actin was prepared by mixing equal amounts (20 μl) of rhodamine-G-actin and biotinylated G-actin (both at 20 μM, 1 mg/mL) in the presence of equimolar concentration (20 μM) of phalloidin in 5 μl G-buffer followed by addition of 5 μl 10x F-buffer to start the polymerization reaction (1 h, RT). 12.5 μl of phalloidin-stabilized, rhodamine-labeled and biotinylated F-actin (16 μM) was mixed with 37.5 μl PBS (for F-actin beads) for a final concentration of 4 μM and incubated for 1 h at RT.

#### Gelsolin treatment of F-actin coupled to microspheres

4 μM biotin/rhodamine-F-actin or biotin/rhodamine-F-actin was diluted 1:4 with PBS and 100 μl was added to 20 μl streptavidin-coated beads (2μm; Polysciences Inc.), which had been washed twice with wash buffer (PBS + 1% BSA), for 30 min on ice. Washed beads were resuspended in wash buffer and sonicated (2 × 2 min) in a water bath sonicator before storage. F-actin-coupled microbeads were resuspended in HBSS containing 1 mM Ca^2+^ and 10 μg/mL sGsn and incubated for 30 min on ice, followed by addition of FLAG-mDNGR-1 reagent. Beads were washed and stained with fluorescent-labeled antibodies including AlexaFluor647-conjugated rat-anti-DNGR-1 antibody (1F6) and AlexaFluor488-conjugated mouse-anti-human gelsolin antibody. Duplicate samples were stained with mouse anti-actin antibody (AC-40) followed by AlexaFluor488-conjugated goat anti-mouse IgG antibody.

#### *In vitro* cross-presentation

cDC1-mediated cross-presentation of bm1OVAMEF and 5555 Braf^V600E^ cells was carried out as described recently (Schulz et al., 2018). Briefly, cells were UV-irradiated (240 mJ/cm^2^) and left for several h in serum-free RPMI1640 medium. 5555 Braf^V600E^ cells were additionally pulsed with OVA (10 mg/mL) for 1 h at 37°C. Dead cells were added to Mutu DCs (1x10^5^/well) at the indicated ratio and cultured in 96-well round-bottom plates at 37°C in RPMI 1640 medium containing 2 mM glutamine, 50 μM 2-mercaptoethanol, 100 units/mL penicillin, 100 μg/mL streptomycin and 2.5% heat-inactivated sGsn-deficient mouse serum. To facilitate dead cell uptake, plates were centrifuged at 1000 rpm for 3 min at the start of the incubation. Pre-activated OT-I T cells (5x10^4^/well) (Hanč et al., 2016b) were added after 4 h and OT-I T cell activation was determined by measuring IFN-γ levels in the supernatant of overnight cultures by ELISA.

#### N. brasiliensis infection

*N. brasiliensis* was a generous gift from Judi Allen (University of Manchester). The parasite was maintained by serial passage through rats, as described previously (Camberis et al., 2003). L3 larvae were extracted from faecal pellets by use of a modified Baermann apparatus and collected in PBS. After at least 3 rounds of washing in sterile PBS, larval numbers were counted and further diluted as needed. Mice were infected subcutaneously with 250 L3 larvae per mouse. For analysis by RT-qPCR, broncho-alveolar lavage fluid (BALF) samples taken at sacrifice were immediately transferred into lysis buffer. Approximately 20 μg tissue from each lung was homogenized using a TissueLyser II (QIAGEN) and clarified using QiaShredder columns (QIAGEN). RNA was extracted using a column-based method (QIAGEN). cDNA synthesis was performed using SuperScript II Reverse Transcriptase (Thermo Fisher Scientific), and random hexamers (Thermo Fischer Scientific). cDNA was then diluted eight times in nuclease-free water and analyzed for transcript presence by qPCR using PowerUp SYBR Green master mix (Thermo Fisher Scientific). Reactions were carried out using QuantStudio 3 or QuantStudio 5 machines (Thermo Fisher Scientific). Primers sequences for qRT-PCR used can be found in Table S4. Relative expression values were calculated from ΔCts using *18S* mRNA as a reference gene. For analysis of BALF cellular content, BALF samples were centrifuged for 8 min at 1400 rpm and the pellet was resuspended in FACS buffer (PBS with 4% FCS, 5 mM EDTA and 0.2% azide), washed once, and then resuspended in PBS for staining. For analysis of total lung leukocyte content, lungs were chopped to small pieces and digested with collagenase IV (200 U/mL) and DNase I (100 mg/mL) for 60 min at 37 þC. Tissue was passed through a 70 μm cell strainer (Falcon) and resuspended in Percoll (GE Healthcare). Leukocytes were enriched by Percoll gradient, washed once with FACS buffer, and resuspended in PBS for staining.

For BALF and lung leukocyte staining, samples were incubated with Fc block for 10-15 min on ice, and subsequently stained for 20-30 min on ice in the dark. Following staining, samples were washed once in PBS and fixed (Nordic-MUbio). Samples were then washed three times and stored at 4 þC in the dark until acquisition on a LSR Fortessa (BD Biosciences). Quantification of total cell numbers by flow cytometry was done using beads (Beckman Coulter). Analysis of data was done in FlowJo. After gating on live, single cells, immune cell populations were defined the following: alveolar macrophages (CD45^+^ CD64^+^ F4/80^+^ CD11c^+^ CD11b^-^), monocytes (CD45^+^ CD64^-^ F4/80^-^ Ly-6G^-^ Ly-6C^+^), dendritic cells (CD45^+^ CD64^-^ F4/80^-^ MHCII^+^ CD11c^+^), neutrophils (CD45^+^ CD64^-^ F4/80^-^ Ly-6G^+^ Ly-6C^+^), eosinophils (CD45^+^ CD64^-^ F4/80^-^ Siglec-F^+^).

Cytokines were measured in undiluted BALF. All cytokine levels, were assessed using cytometric bead array according to the manufacturer’s instruction (BD Biosciences).

#### OVA-specific antibody measurements

Mouse serum was prepared from blood collected by cardiac puncture, immediately placed into clotting-activator containing microtubes (1.1 mL Z-gel, Sarstedt), allowed to coagulate for 30 min at room temperature and centrifuged (10,000 rpm, 2 min). OVA-specific antibodies were measured in the serum of tumor-bearing mice by ELISA. Ten-fold serial dilutions of serum samples were added to Iimmunoplates coated with ovalbumin (100 μg/mL) and OVA-specific IgG was detected using a mouse-specific anti-IgG antibody. EC_50_ values for each serum sample were calculated from the titration curves using an algorithm supplied by the ELISA plate reader (SoftMax Pro) and mice whose titer could not be calculated or was above 0.15 were excluded from the analysis.

#### Influenza A virus infection

Influenza A virus (X31) was a gift from Andreas Wack (The Francis Crick Institute). Mice were infected intranasally with 2.4x10^3^ TCID_50_ per mouse. Lungs were harvested 40 days after infection and single cell suspensions were prepared by collagenase IV/DNase I digestion (see below). Influenza A - specific CD8^+^ memory T cells were analyzed by FACS following staining of lung cells with D^b^-NP_366-374_ pentamer and antibodies against CD8, CD103, CD62L and CD44. The percentage of pentamer^+^ CD103^-^ effector memory T cells were analyzed after gating on CD8^+^CD44^+^CD62L^-^ cells. Quantification of total cell numbers by flow cytometry was done using beads (Beckman Coulter). Analysis of data was done in FlowJo.

#### Analysis of tumor tissue, tdLNs and lymphoid organs

Tumors and tumor draining lymph nodes (tdLN) were excised at the indicated days after cell transplantation. Tumor mass of individual tumors was determined using a microscale. For subsequent analysis by flow cytometry, tumors and tdLN were cut into pieces and digested with collagenase IV (200 U/mL) and DNase I (100 μg/mL) for 30 min at 37°C. Tissue was passed through a 70 μm cell strainer (Falcon), washed with FACS buffer (PBS with1%FCS and 2mM EDTA) and cells were incubated with Fc block (CD16/32, clone 2.4G2, BD Biosciences) for 10 min in 4°C before proceeding with antibody mediated staining.

For the *ex vivo* analysis of T cells, cell suspensions were stained with PE-conjugated H-2K^b^/SIINFEKL pentamer (ProImmune) for 15 min at RT. Cells washed and stained with LIVE/DEAD Fixable Blue Dead Cell dye (ThermoFischer Scientific) according to manufacturer’s protocol and subsequently stained with various lineage specific antibodies: V500-CD45 (30-F11, BD Biosciences, 1:100 dilution), APC-CD3e (145-2C11, 1:100 dilution), APC-Cy7-CD8α (53-6.7, Biolegend, 1:200 dilution). Cells were fixed (Nordic-MUbio) prior to analysis. Quantification of total cell numbers by flow cytometry was done using beads (Beckman Coulter).

For the *ex vivo* analysis of cDC1 cells and for phenotypic characterization of *sGsn*^*−/−*^ mice, cells from primary and secondary lymphoid tissues were digested as before and stained with LIVE/DEAD Fixable Blue Dead Cell dye (ThermoFischer Scientific) according to manufacturer’s protocol and subsequently stained in the presence of various lineage specific antibodies: BV421-XCR-1 (ZET, Biolegend, 1:100 dilution), BV605-Ly6C (HK1.4, Biolegend, 1:100 dilution), BV605-CD8α (53-6.7, Biolegend, 1:200 dilution), BV605-CD45.2 (104, Biolegend, 1:200 dilution), BV650-B220/CD45R (RA3-6B2, Biolegend, 1:200 dilution), BV711-CD45.2 (104, Biolegend, 1:200 dilution), BV421-CD206 (C068C2, Biolegend, 1:100 dilution) FITC-CD11b (M1/70, BD Biosciences, 1:100 dilution), PerCP-Cy5.5-GR-1 (RB6-8C5, Biolegend, 1:100 dilution), BV711-CD86 (GL1, BD Biosciences, 1:100 dilution), PerCP-Cy5.5-CD4 (RM4-5, BD Biosciences, 1:200 dilution), PerCP-Cy5.5-CD103 (2E7, Biolegend, 1:100 dilution), PE-NK1.1 (PK136, BD Biosciences, 1:100 dilution), FITC-NK1.1 (PK136, Biolegend, 1:100 dilution), BV421-CD19 (6D5, Biolegend, 1:200 dilution) AlexaFluor700-CD19 (6D5, Biolegend, 1:100 dilution), APCeF780-CD3e (145-2C11, E-Bioscience, 1:100 dilution), PE-CD4 (RM4-5, BD Biosciences, 1:200 dilution), PE-Cy7-TCR-delta (GL3, Biolegend, 1:100 dilution), PE-Cy7-CD64 (X54-5/7., Biolegend, 1:100 dilution), AlexaFluor647-Sirpα (P84, Biolegend, 1:100 dilution), APC-CD8α (53-6.7, BD Biosciences, 1:200 dilution), AlexaFluor700-MHC-II(I-A/I-E) (M5/114.15.2, E-Bioscience, 1:100 dilution), APCeFluor780-CD11c (N418, E-Bioscience, 1:100 dilution), APC-Cy7-TCRbeta (H57-597, Biolegend, 1:200 dilution). Cells were fixed (Nordic-MUbio) prior to analysis. For intracellular staining the samples were stained in permeabilization buffer using the following antibodies for defining T cell effector subset: BV421-GATA-3 (16E10A23, Biolegend, 1:100 dilution), BV650-RORγt (Q31-378, E-Biosciences, 1:100 dilution), PE-FOXP3 (FJK-16S, E-Bioscience, 1:100 dilution), BV711-Tbet (Apr-46, BD-Horizon, 1:100 dilution). Fixation and permeabilization were perfomed using the Fixation/Permeabilisation buffer-Foxp3 Kit (E-Biosciences) according to the manufacturer’s protocol. Quantification of total cell numbers by flow cytometry was done using beads (Beckman Coulter). Samples were acquired on a Fortessa X20 B (BD Biosciences). Data were analyzed using FlowJo software. Gating strategies are provided as supplemental items (Data S1–S3).

#### *Ex vivo* cross-presentation assay

Inguinal and axillary tumor draining lymph nodes (tdLNs) of WT, *sGsn*^*−/−*^ or *sGsn*^*−/−*^ *Clec9a*^*gfp/gfp*^ mice at day 14 post-tumor (B16F10 LA-OVA-mCherry) were digested as before and stained with LIVE/DEAD Fixable Aqua Dead Cell dye (ThermoFischer Scientific) according to manufacturer’s protocol and subsequently stained with the following antibodies:

BV421-CD11c (N418, Biolegend 1:200 dilution), FITC-MHCII(I-A/I-E) (M5/114.15.2, E-Bioscience, 1:200 dilution), PerCP-Cy5.5-B220/CD45R (RA3-6B2, Biolegend, 1:200 dilution), BV785-XCR-1 (ZET, Biolegend, 1:100 dilution), APC/Fire 750-Sirpα (P84, Biolegend,1:100 dilution). Migratory cDC1 (live B220^-^ CD11c^+^ MHCII^High^ Sirpα^-^ XCR-1^+^) were sorted using a FACSAria Fusion sorter. Spleen and lymph nodes of OT-I x *Rag1*^*−/−*^ were isolated and enriched for naive OT-I by the EasySep™ Mouse Naive CD8^+^ T cell isolation kit (STEMCELL Technologies) and subsequent were labeled with 1 μM VPD450 cell division dye (BD Biosciences) for 15 min at 37°C according to manufacturer’s protocol. 10^3^ migratory cDC1 sorted from tdLN were co-cultured with 2x10^4^ labeled naive OT-I in 96-well V-bottom plate for 72 h at 37°C. After 3 days the cell mixture was stained with LIVE/DEAD Fixable Blue Dead Cell dye (ThermoFischer Scientific) and subsequent with FITC-CD8α (53-6.7, BD Biosciences, 1:200 dilution) and APC-CD44 (IM7, BD Biosciences, 1:200 dilution) and analyzed by flow cytometry. Cells were fixed (Nordic-MUbio) prior to analysis. The proportion of proliferated (VPD450 dye dilution) activated OT-I (live CD8α^+^ CD44^High^) was calculated as a surrogate of cross-presentation.

#### DNGR-1 binding reporter assay

A reporter assay for DNGR-1 binding has been described previously (Ahrens et al., 2012; Sancho et al., 2009). Briefly, BWZ-mDNGR-1-ζ-chain cells were plated in 96 well plates (1x10^5^ cells/well) in the presence of added stimuli as indicated. Stimulation of reporter cells was performed in RPMI 1640 medium containing 2 mM glutamine, 50 μM 2-mercaptoethanol, 100 units/mL penicillin, 100 μg/mL streptomycin and 2.5% sGsn-deficient mouse serum. After overnight culture, cells were washed once in PBS and LacZ activity was measured by lysing cells in chlorophenol red-β-D-galactopyranoside (CPRG, Roche)-containing buffer. 1-4 h later absorbance (O.D. 595 nm using O.D. 655 nm as a reference) was measured .

#### Bioinformatic analysis of human tissues and cancer patient data

Normalized read counts for gelsolin isoform expression were downloaded from the Genotype-Tissue Expression (GTEx) resourse Biobank [https://gtexportal.org]. Raw count data for each TCGA dataset was downloaded from [https://gdac.broadinstitute.org/] and normalized using DESEQ2 (Love et al., 2014). Tumor only samples were ranked using normalized GSN expression. Differential expression between low and high expressing GSN groups was determined using the Wald’s test. The Wald’s statistic was used to rank genes using Preranked GSEA (version 2.2.3) (Subramanian et al., 2005) and statistically significant pathways identified from the c2 pathway genesets [MSigdb] (Liberzon et al., 2011). Overall survival analyses were performed for the high and low expression ranked values for cytoplasmic (*cGSN*: uc011lyh.2, GenBank: NM_001258029 and uc010mvu.2, GenBank: NM_001127663) and secreted (*sGSN*: uc004ble.1, GenBank: NM_198252) *GSN* isoforms and plotted for Kaplan-Meier curves using GraphPad Prism (GraphPad). using the REACTOME database. MHC class I (cross)-presentation, cell death and immunity gene signatures can be found in REACTOME pathway database [https://reactome.org]. cDC1 gene signature is composed of the following genes: *CLEC9A, XCR1, CKNK, BATF3* (Böttcher et al., 2018). Effector CD8 T cell gene signature is composed of the following genes: *CD3, CD8A, CXCL10, CXCL9, GZMA, GZMB, IFNG, PRF1* (Böttcher et al., 2018; Mariathasan et al., 2018). Total tumor mutational counts, mutational counts for F-actin binding proteins (Table S2) and microtubule binding proteins (Table S3) for each TCGA dataset were downloaded from the TCGA Pan-Cancer Atlas [https://www.cbioportal.org].

### Quantification and statistical analysis

All statistical analyses were performed using GraphPad Prism software (GraphPad). Statistical significance between two groups was determined using an unpaired two-tailed Student’s t test. Statistical analyses for two or more groups were done by one or two way ANOVA followed by Bonferroni multiple-comparison post hoc correction. One-way ANOVA was used to compare average means of two or more groups obtained in a single time point. Two-way ANOVA was used to compare average means of two or more dose response curves for *in vitro* assays and individual means per time point for tumor growth profiles. The Log-rank (Mantel-Cox) test was used to determine statistical significance for overall survival in cancer patient data from TCGA. In the gene-enrichment analysis using genes were ranked by the Wald’s test false discovery rate (FDR)-adjusted p were calculated. Auto-antibody scores were compared using two-tailed Wilcoxon matched-pairs signed rank test. Pearson’s correlation coefficient (r) was calculated as a measure of the strength of the association between the expression values of two genes or gene signatures. Finally, two- tailed chi-square was used to determine any significant differences in frequencies of different clinical parameters between two groups. Data are shown as mean ± SD or mean ± SEM as indicated in the figure legends. Significance was assumed with ^∗^p < 0.05; ^∗∗^p < 0.01; ^∗∗∗^p < 0.001, ^∗∗∗∗^p < 0.0001.
