# Supplementary material for: Secreted gelsolin inhibits DNGR-1-dependent cross-presentation and cancer immunity
Source: Cell. 2021 Jul 22;184(15):4016–4031.e22. doi: 10.1016/j.cell.2021.05.021 (PMC8320529; doi:10.1016/j.cell.2021.05.021)

Cell, Volume *184*

Supplemental information

Secreted gelsolin inhibits DNGR-1-dependent cross-presentation and cancer immunity

Evangelos Giampazolias, Oliver Schulz, Kok Haw Jonathan Lim, Neil C. Rogers, Probir Chakravarty, Naren Srinivasan, Oliver Gordon, Ana Cardoso, Michael D. Buck, Enzo Z. Poirier, Johnathan Canton, Santiago Zelenay, Stefano Sammicheli, Natalia Moncaut, Sunita Varsani-Brown, Ian Rosewell, and Caetano Reis e Sousa

Table S1. Distribution of clinical parameters (age, sex and tumour stage) between *sGSN*^{Low} and *sGSN*^{High} groups in human tumours, related to Figure 6

TCGA dataset

LIHC

**AGE** (years old)	Group	10_19	20_29	30_39	40_49	50_59	60_69	70_79	80_89
Chi-Square test	*sGSN* High	1	4	7	9	17	21	12	2
p value = 0.79 ns	*sGSN* Low	0	3	3	10	18	27	12	1

**SEX**	Group	Female	Male
Chi-Square test	*sGSN* High	28	46
p value = 0.38 ns	*sGSN* Low	22	52

**STAGE**	Group	0	1	2	3	4
Chi-Square test	*sGSN* High	6	28	19	20	1
p value = 0.30 ns	*sGSN* Low	1	34	22	16	1

HNSC

**AGE** (years old)	Group	10_19	20_29	30_39	40_49	50_59	60_69	70_79	80_89
Chi-Square test	*sGSN* High	1	0	2	14	30	39	9	9
p value = 0.75 ns	*sGSN* Low	0	0	1	13	33	39	13	5

**SEX**	Group	Female	Male
Chi-Square test	*sGSN* High	33	71
p value = 0.15 ns	*sGSN* Low	23	81

**STAGE**	Group	0	1	2	3	4
Chi-Square test	*sGSN* High	1	4	17	20	62
p value = 0.27 ns	*sGSN* Low	6	2	12	20	64

STAD

**AGE** (years old)	Group	30_39	40_49	50_59	60_69	70_79	80_89
Chi-Square test	*sGSN* High	1	4	12	12	8	4
p value =0.49 ns	*sGSN* Low	0	5	8	10	15	3

**SEX**	Group	Female	Male
Chi-Square test	*sGSN* High	15	26
p value = 0.82 ns	*sGSN* Low	17	24

**STAGE**	Group	0	1	2	3	4
Chi-Square test	*sGSN* High	1	4	15	15	6
p value = 0.22 ns	*sGSN* Low	1	7	9	22	2

Table S2. List of human genes encoding F-actin binding proteins, related to Figure 7

F-actin_binding_proteins	Human_gene_symbol	Full_human_gene_name
1	*ACTR2*	Actin-related protein 2
2	*ACTR3*	Actin-related protein 3
3	*ACTN1*	Alpha-Actinin
4	*ANLN*	Anillin
5	*CAPZA1*	Capping actin protein of muscle Z-line subunit alpha 1
6	*CAPZA2*	Capping actin protein of muscle Z-line subunit alpha 2
7	*CAPZB*	Capping actin protein of muscle Z-line subunit beta
8	*CARMIL1*	Capping protein regulator and myosin 1 linker 1
9	*CDC42*	Cell division control protein 42 homolog
10	*CFL1*	Cofilin 1
11	*CFL2*	Cofilin 2
12	*EZR*	Ezrin
13	*FSCN1*	Fascin
14	*FLNA*	Filamin A
15	*FLNB*	Filamin B
16	*FMN1*	Formin-1
17	*FMN2*	Formin-2
18	*GSN*	Gelsolin
19	*CCDC88A*	Girdin
20	*CAPG*	Macrophage-capping protein
21	*MSN*	Moesin
22	*MYH1*	Myosin heavy chain 1
23	*MYH10*	Myosin heavy chain 10
24	*MYH11*	Myosin heavy chain 11
25	*MYH13*	Myosin heavy chain 13
26	*MYH14*	Myosin heavy chain 14
27	*MYH15*	Myosin heavy chain 15
28	*MYH16*	Myosin heavy chain 16
29	*MYH2*	Myosin heavy chain 2
30	*MYH3*	Myosin heavy chain 3
31	*MYH4*	Myosin heavy chain 4
32	*MYH6*	Myosin heavy chain 6
33	*MYH7*	Myosin heavy chain 7
34	*MYH8*	Myosin heavy chain 8
35	*MYH9*	Myosin heavy chain 9
36	*MYO3A*	Myosin IIIA
37	*MYO3B*	Myosin IIIB
38	*MYO9A*	Myosin IXA
39	*MYO9B*	Myosin IXB
40	*MYL5*	Myosin light chain 5
41	*MYL1*	Myosin light chain 1
42	*MYL2*	Myosin light chain 2
43	*MYL3*	Myosin light chain 3
44	*MYL4*	Myosin light chain 4
45	*MYL6*	Myosin light chain 6
46	*MYL6B*	Myosin light chain 6B
47	*MYL7*	Myosin light chain 7
48	*MYL9*	Myosin light chain 9
49	*MYLK2*	Myosin light chain kinase 2, skeletal/cardiac muscle
50	*MYLK*	Myosin light chain kinase, smooth muscle
51	*MYLIP*	Myosin regulatory light chain interacting protein
52	*MYO5A*	Myosin VA
53	*MYO5B*	Myosin VB
54	*MYO5C*	Myosin VC
55	*MYO6*	Myosin VI
56	*MYO7A*	Myosin VIIA
57	*MYO7B*	Myosin VIIB
58	*MYO10*	Myosin X
59	*MYO15A*	Myosin XVA
60	*MYO18A*	Myosin XVIIIA
61	*MYO18B*	Myosin XVIIIB
62	*MYO1A*	Myosin-IA
63	*MYO1B*	Myosin-IB
64	*MYO1C*	Myosin-IC
65	*MYO1D*	Myosin-ID
66	*MYO1E*	Myosin-IE
67	*MYO1F*	Myosin-IF
68	*MYO1G*	Myosin-IG
69	*MYO1H*	Myosin-IH
70	*MYLL1*	Myosin, light chain, cardiac muscle-Like 1
71	*DIAPH1*	Protein diaphanous homolog 1
72	*RDX*	Radixin
73	*SCIN*	Scinderin
74	*SPTA1*	Spectrin alpha, erythrocytic 1
75	*SPTAN1*	Spectrin alpha, nonerythrocytic 1
76	*SPTB*	Spectrin beta, erythrocytic
77	*SPTBN1*	Spectrin beta, nonerythrocytic 1
78	*SPTBN2*	Spectrin beta, nonerythrocytic 2
79	*SPTBN4*	Spectrin beta, nonerythrocytic 4
80	*SPIRE1*	Spire type actin nucleation factor 1
81	*SPIRE2*	Spire type actin nucleation factor 2
82	*TMOD1*	Tropomodulin-1
83	*TMOD2*	Tropomodulin-2
84	*TMOD3*	Tropomodulin-3
85	*TPM1*	Tropomyosin alpha-1 chain
86	*TPM3*	Tropomyosin alpha-3 Chain
87	*TPM4*	Tropomyosin alpha-4 chain
88	*TPM2*	Tropomyosin beta chain
89	*VASP*	Vasodilator-stimulated phosphoprotein
90	*VIL1*	Villin1

Table S3. List of human genes encoding microtubule binding proteins, related to Figure 7

Microtubule_binding_proteins	Human_gene_symbol	Full_human_gene_name
1	*ASPM*	Abnormal spindle-like microcephaly-associated protein
2	*CEP55*	Centrosomal protein of 55 kDa
3	*CKAP5*	Cytoskeleton-associated p5otein
4	*CLIP1*	CAP-Gly domain containing linker protein 1
5	*CLIP2*	CAP-Gly domain containing linker protein 2
6	*DCTN1*	Dynactin subunit 1
7	*DCTN2*	Dynactin subunit 2
8	*DLGAP5*	Disks large-associated protein 5
9	*DNAH1*	Dynein axonemal heavy chain 1
10	*DNAH10*	Dynein axonemal heavy chain 10
11	*DNAH11*	Dynein axonemal heavy chain 11
12	*DNAH12*	Dynein axonemal heavy chain 12
13	*DNAH14*	Dynein axonemal heavy chain 14
14	*DNAH17*	Dynein axonemal heavy chain 17
15	*DNAH2*	Dynein axonemal heavy chain 2
16	*DNAH3*	Dynein axonemal heavy chain 3
17	*DNAH5*	Dynein axonemal heavy chain 5
18	*DNAH6*	Dynein axonemal heavy chain 6
19	*DNAH7*	Dynein axonemal heavy chain 7
20	*DNAH8*	Dynein axonemal heavy chain 8
21	*DNAH9*	Dynein axonemal heavy chain 9
22	*DNAI1*	Dyneins intermediate chain 1
23	*DNAI2*	Dyneins intermediate chain 2
24	*DNAL1*	Dynein light intermediate chains 2
25	*DNAL4*	Dynein light intermediate chains 4
26	*DNALI1*	Dynein light intermediate chains 1
27	*DYNC1H1*	Dynein cytoplasmic 1 heavy chain 1
28	*DYNC1LI1*	Dynein cytoplasmic 1 light intermediate chain 1
29	*DYNC1LI2*	Dynein cytoplasmic 1 light intermediate chain 2
30	*DYNC2LI1*	Dynein cytoplasmic 2 light intermediate chain 1
31	*DYNLL1*	Dynein light chain 1, cytoplasmic
32	*DYNLL2*	Dynein light chain 2, cytoplasmic
33	*DYNLRB1*	Dynein light chain roadblock-type 1
34	*DYNLRB2*	Dynein light chain roadblock-type 2
35	*DYNLT1*	Dynein light chain Tctex-type 1
36	*DYNLT3*	Dynein light chain Tctex-type 3
37	*KATNA1*	Katanin p60 ATPase-containing subunit A1
38	*KATNB1*	Katanin p60 ATPase-containing subunit B1
39	*KIF11*	Kinesin family member 11
40	*KIF12*	Kinesin family member 12
41	*KIF13A*	Kinesin family member 13A
42	*KIF13B*	Kinesin family member 13B
43	*KIF14*	Kinesin family member 14
44	*KIF15*	Kinesin family member 15
45	*KIF16B*	Kinesin family member 16B
46	*KIF17*	Kinesin family member 17
47	*KIF18A*	Kinesin family member 18A
48	*KIF19*	Kinesin family member 19
49	*KIF1A*	Kinesin family member 1A
50	*KIF1B*	Kinesin family member 1B
51	*KIF1C*	Kinesin family member 1C
52	*KIF20A*	Kinesin family member 20A
53	*KIF20B*	Kinesin family member 20B
54	*KIF21A*	Kinesin family member 21A
55	*KIF21B*	Kinesin family member 21B
56	*KIF22*	Kinesin family member 22
57	*KIF23*	Kinesin family member 23
58	*KIF24*	Kinesin family member 24
59	*KIF25*	Kinesin family member 25
60	*KIF26A*	Kinesin family member 26A
61	*KIF26B*	Kinesin family member 26B
62	*KIF27*	Kinesin family member 27
63	*KIF2A*	Kinesin family member 2A
64	*KIF2C*	Kinesin family member 2C
65	*KIF3A*	Kinesin family member 3A
66	*KIF3B*	Kinesin family member 3B
67	*KIF3C*	Kinesin family member 3C
68	*KIF4A*	Kinesin family member 4A
69	*KIF4B*	Kinesin family member 4B
70	*KIF5A*	Kinesin family member 5A
71	*KIF5B*	Kinesin family member 5B
72	*KIF5C*	Kinesin family member 5C
73	*KIF6*	Kinesin family member 6
74	*KIF7*	Kinesin family member 7
75	*KIF9*	Kinesin family member 9
76	*KIFAP3*	Kinesin-2 associated protein
77	*KIFC1*	Kinesin family member C1
78	*KIFC2*	Kinesin family member C2
79	*KIFC3*	Kinesin family member C3
80	*KLC1*	Kinesin light chain 1
81	*KLC2*	Kinesin light chain 2
82	*KLC3*	Kinesin light chain 3
83	*KLC4*	Kinesin light chain 4
84	*MAP1A*	Microtubule-associated protein 1A
85	*MAP1B*	Microtubule-associated protein 1B
86	*MAP2*	Microtubule-associated protein 2
87	*MAP4*	Microtubule-associated protein 4
88	*MAP6*	Microtubule-associated protein 6
89	*MAP7*	Microtubule-associated protein 7
90	*MAP9*	Microtubule-associated protein 9
91	*MAPRE1*	Microtubule-associated protein RP/EB family member 1
92	*MAPRE2*	Microtubule-associated protein RP/EB family member 2
93	*MAPRE3*	Microtubule-associated protein RP/EB family member 3
94	*MAPT*	Microtubule associated protein tau
95	*PAFAH1B1*	Platelet-activating factor acetylhydrolase IB subunit alpha
96	*PLEC*	Plectin
97	*TUBG1*	Tubulin gamma 1

Table S4. List of primers for RT-qPCR, related to STAR Methods, Key Resources Table, Oligonucleotides

Mouse Primers	Name	Sequence
**RT-qPCR**	*Ccl24* Fwd	CCAAGAAGGGCCATAAGATCTG
	Ccl24 Rev	GCCCCTTTAGAAGGCTGGTT
	Ccl3 Fwd	ACTGCCCTTGCTGTTCTTCT
	Ccl3 Rev	CTGCCGGTTTCTCTTAGTCA
	Retnla Fwd	GACTGCTACTGGGTGTGCTT
	Retnla Rev	GCTGGGTTCTCCACCTCTTC
	Chil4 Fwd	AAGGCTGCTACTCACTTCCAC
	Chil4 Rev	TCCAGCACTAACAGTAGGGTCA
	Arg1 Fwd	GGAATCTGCATGGGCAACCTGTGT
	Arg1 Rev	AGGGTCTACGTCTCGCAAGCCA
	IL-33 Fwd	TGAGACTCCGTTCTGGCCTC
	IL-33 Rev	CTCTTCATGCTTGGTACCCGAT
	Col1a2 Fwd	GCAGGTTCACCTACTCTGTCCT
	Col1a2 Rev	CTTGCCCCATTCATTTGTCT
	Col3a1 Fwd	CTGGAGAACCTGGTGCAAAT
	Col3a1 Rev	CCTCGGAAGCCACTAGGAC
	Acta2 Fwd	ACTGGGACGACATGGAAAAG
	Acta2 Rev	GTTCAGTGGTGCCTCTGTCA
	Areg2 Fwd	GGTCTTAGGCTCAGGCCATTA
	Areg2 Rev	CGCTTATGGTGGAAACCTCTC
	Nippo_Actin Fwd	ACGACGTGGCAGCTCTCGTTGTGG
	Nippo_Actin Rev	GGTGCTTCGGTCAGCAGCACGGG
	18S RNA Fwd	CGCCGCTAGAGGTGAAATTCT
	18S RNA Rev	CGAACCTCCGACTTTCGTTCT

Data S1. Gating strategy items, related to STAR Methods, Method details, Analysis of tumour tissue, tdLNs and lymphoid organs

Thymus-Lymphoid panel (Figures S1B, D)

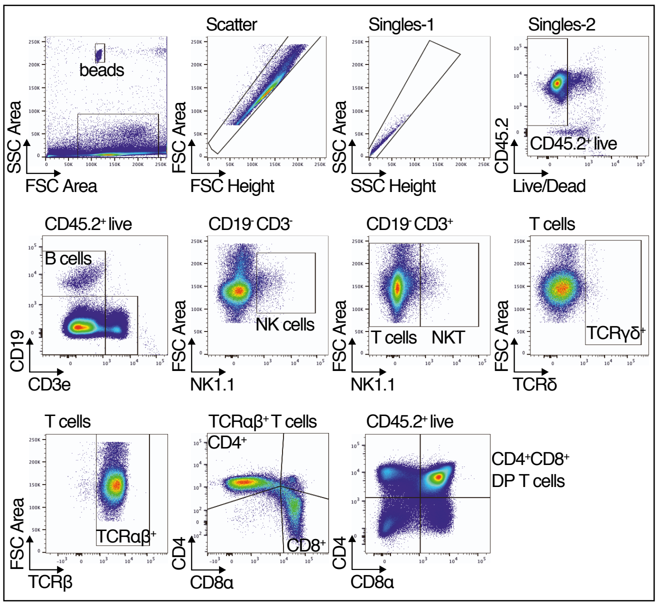

Thymus-Myeloid panel (Figure S1G)

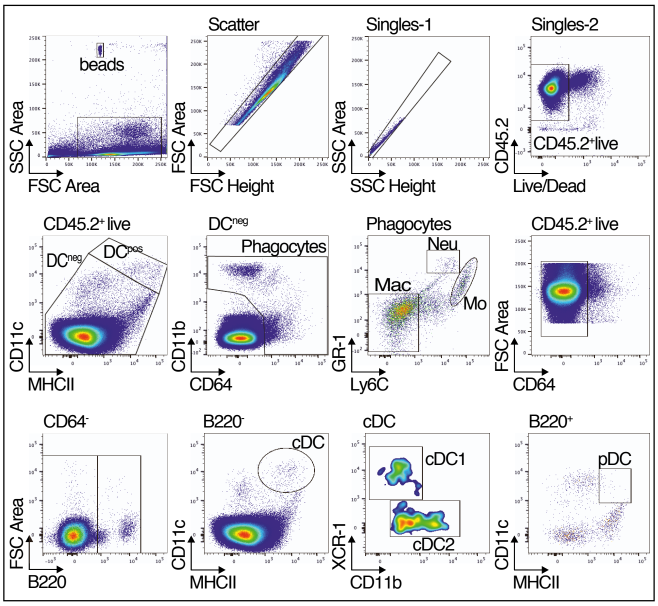

Spleen-Lymphoid panel (Figures S1B, E)

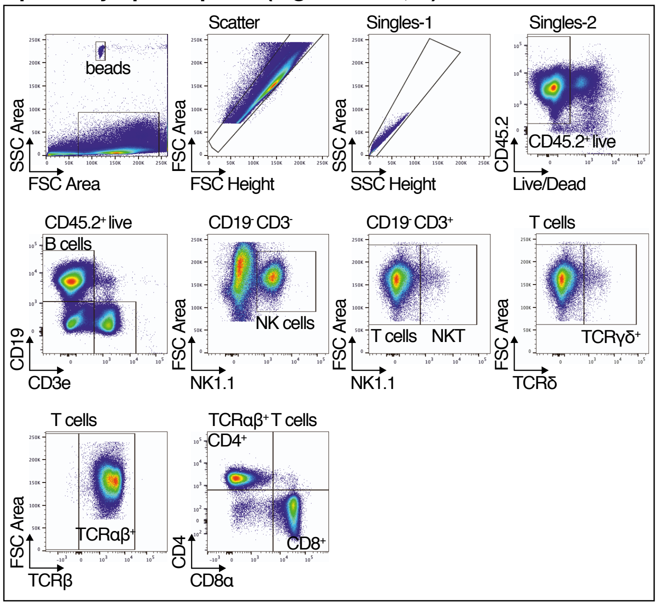

Spleen-Myeloid panel (Figure S1H)

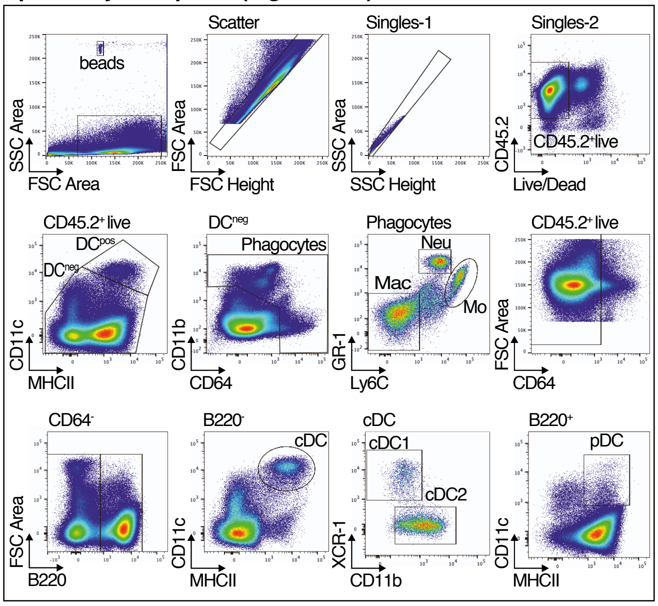

iLN-Lymphoid panel (Figures S1B, F)

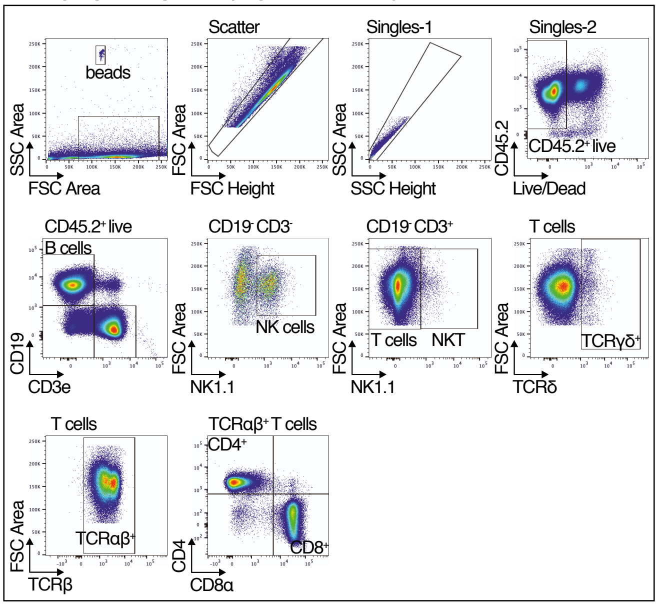

iLN-Myeloid panel (Figure S1I)

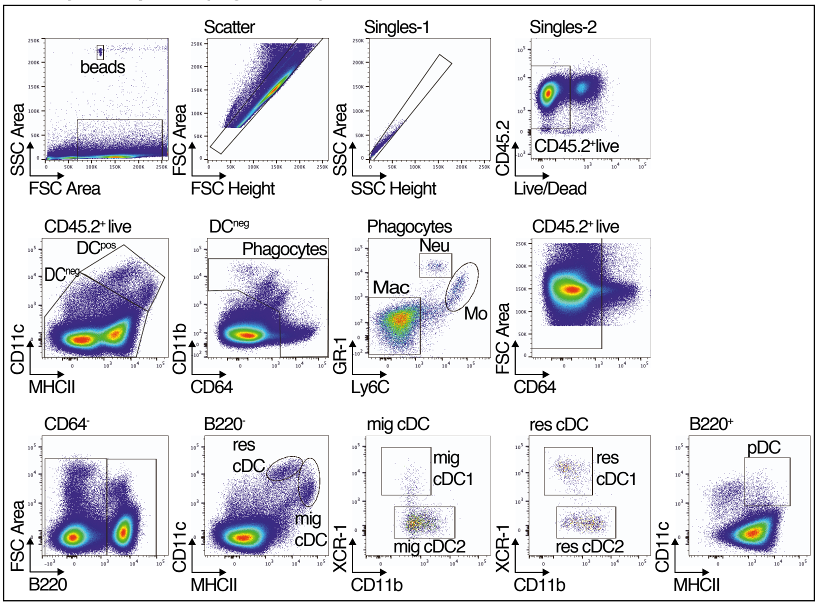

Data S2. Gating strategy items, related to Figure 4

B16 LA-OVA-mCherry tumours-Myeloid panel (Figures 4A, C)

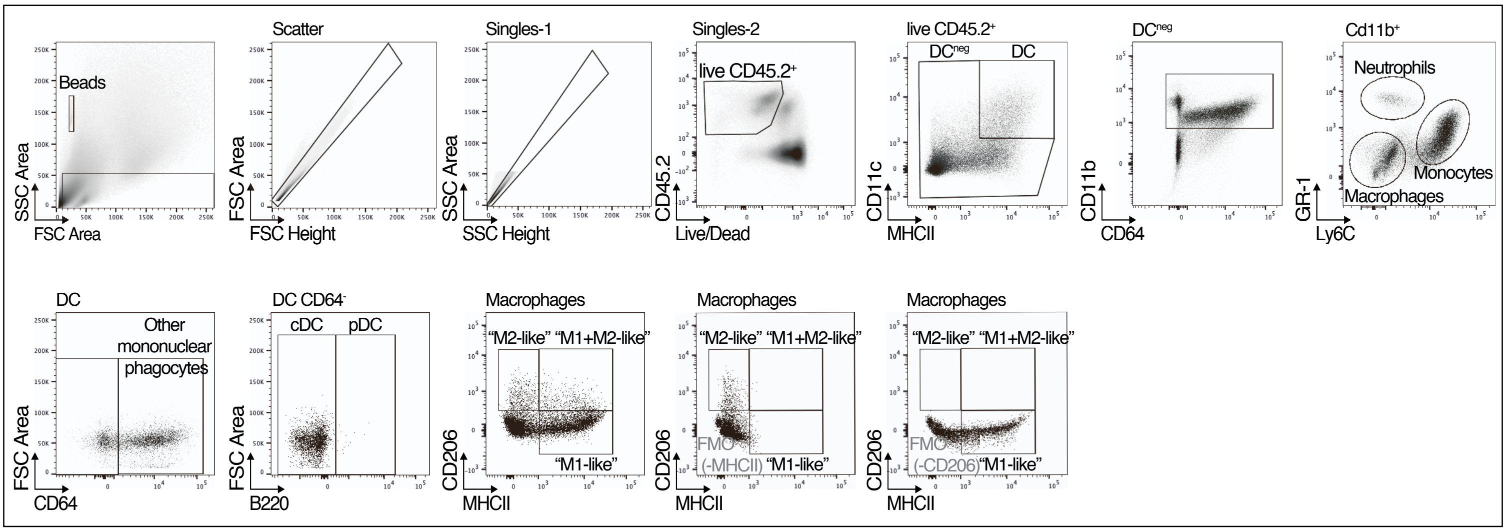

B16 LA-OVA-mCherry tumours-Lymphoid panel (Figures 4B, E)

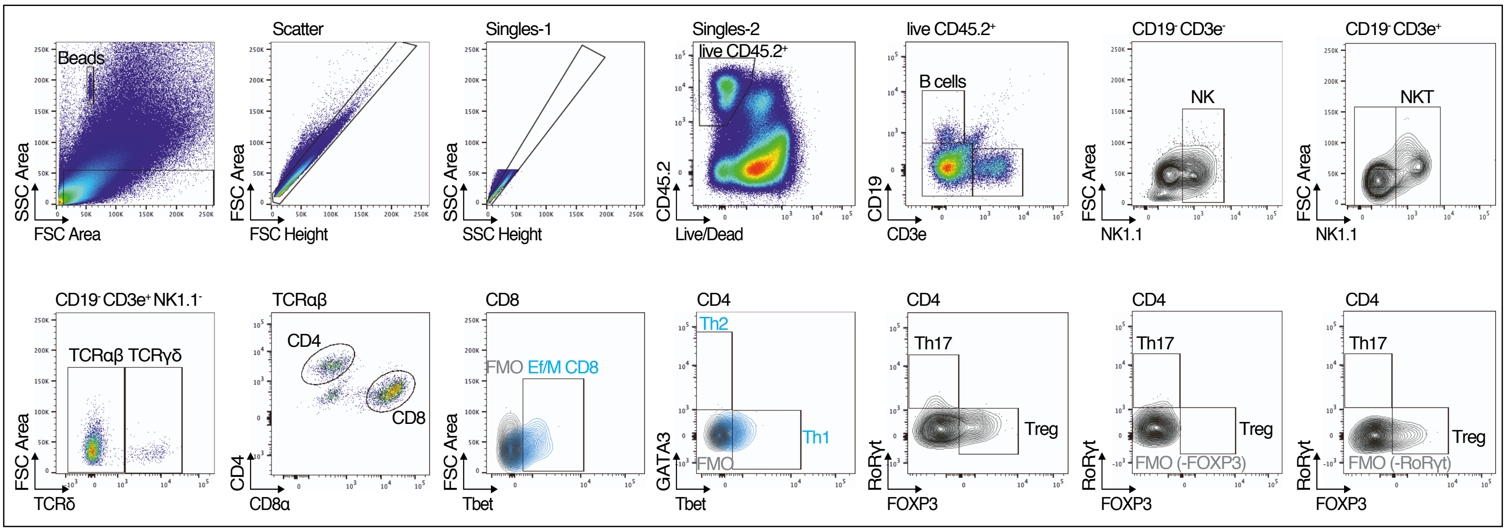

B16 LA-OVA-mCherry tumours + tdLN-DCpanel (Figures 4D and 5B)

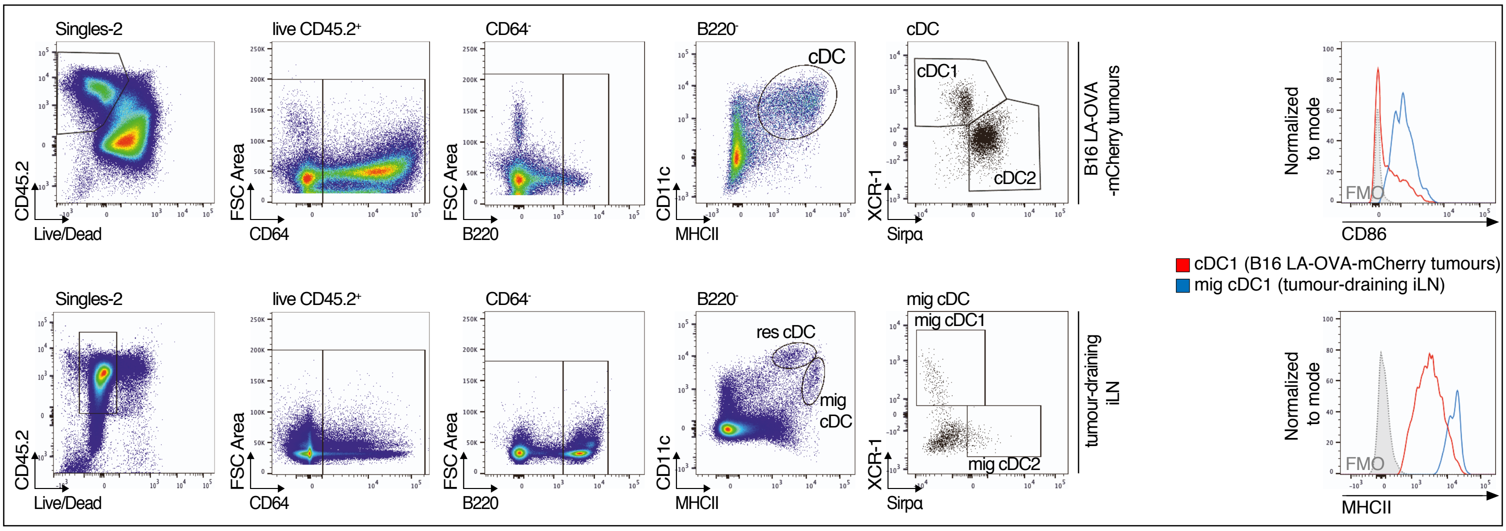

Data S3. Gating strategy items, related to Figure 5 and Supplemental Figure 4

B16 LA-OVA-mCherry tumours-DC panel (Figures 5A and S4C)

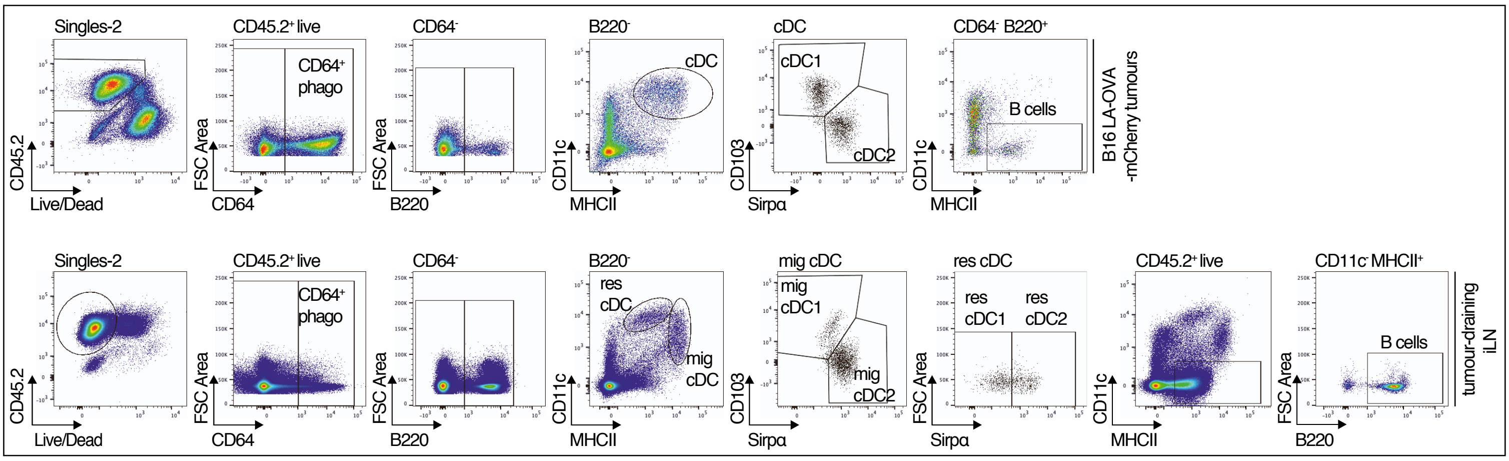

MCA-205 LA-OVA-mCherry tumours-DC panel (Figures S4D, G and H)

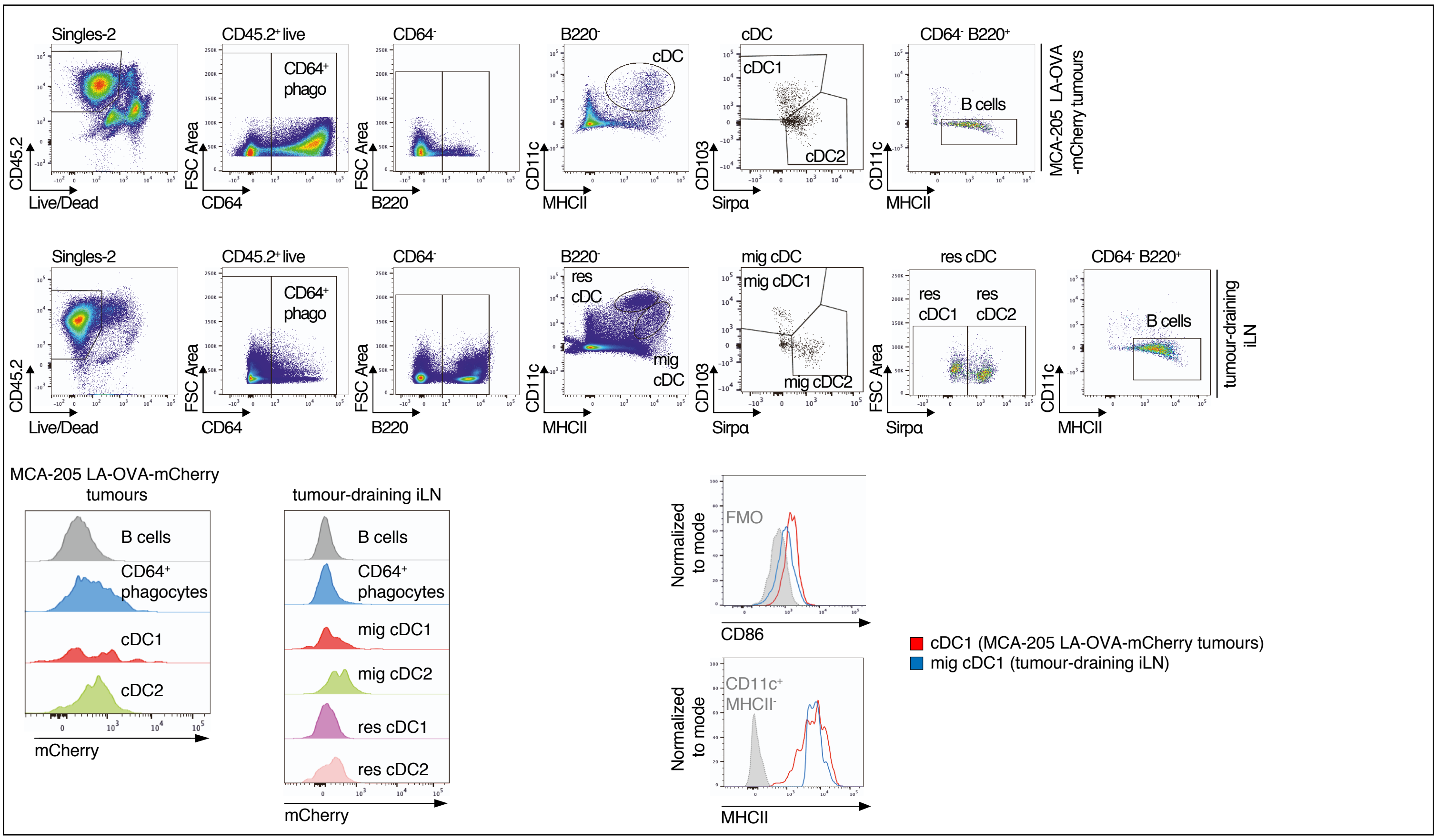

tumour draining axillary and inguinal LN (of B16 LA-OVA-mCherry tumours)-DC panel-FACS sorting (Figures 5C and S4I, J)

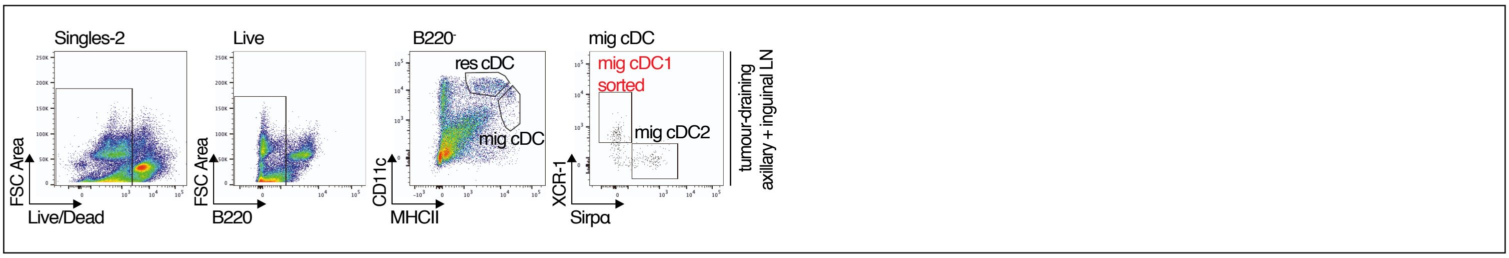

Supplement: Document S1. Tables S1–S4 and Data S1–S3 [file mmc1.pdf]
